# Physical, Thermal and Biological Properties of Yellow Dyes with Two Azodiphenylether Groups of Anthracene

**DOI:** 10.3390/molecules25235757

**Published:** 2020-12-06

**Authors:** Carla Alice Carabet, Anca Moanță, Ion Pălărie, Gabriela Iacobescu, Andrei Rotaru, Marian Leulescu, Mariana Popescu, Petre Rotaru

**Affiliations:** 1 Department of Physics, Faculty of Sciences, University of Craiova, Str. A.I. Cuza, Nr. 13, 200585 Craiova, Romania; alicecarla.benis@gmail.com (C.A.C.); palarie_i@yahoo.com (I.P.); gabrielaiacobescu@yahoo.com (G.I.); marianleulescu@yahoo.com (M.L.); petrerotaru@yahoo.com (P.R.); 2 Department of Chemistry, Faculty of Sciences, University of Craiova, Str. AI Cuza, Nr. 13, 200585 Craiova, Romania; moantaanca@yahoo.com; 3 Department of Biology and Environmental Engineering, Faculty of Horticulture, University of Craiova, Str. A.I. Cuza, Nr. 13, 200585 Craiova, Romania; 4 Institute of Physical Chemistry “Ilie Murgulescu”, Department of Chemical Thermodynamics, Romanian Academy, Splaiul Independentei, Nr. 202, 060021 Bucharest, Romania; 5 Faculty of Pharmacy, University of Medicine and Pharmacy of Craiova, Petru Rareş Street, Nr. 2, 200349 Craiova, Romania; m_tatucu@yahoo.com

**Keywords:** azodiphenylethers, biological properties, bis-azo compounds of anthracene, electronic laser fluorescence, morphologic study, optical anisotropy, thermal analysis and calorimetry, yellow azoic dyes

## Abstract

Two yellow bis-azo dyes containing anthracene and two azodiphenylether groups (BPA and BTA) were prepared, and an extensive investigation of their physical, thermal and biological properties was carried out. The chemical structure was confirmed by the FTIR spectra, while from the UV–Vis spectra, the quantum efficiency of the laser fluorescence at the 476.5 nm was determined to be 0.33 (BPA) and 0.50 (BTA). The possible transitions between the energy levels of the electrons of the chemical elements were established, identifying the energies and the electronic configurations of the levels of transition. Both crystals are anisotropic, the optical phenomenon of double refraction of polarized light (birefringence) taking place. Images of maximum illumination and extinction were recorded when the crystals of the bis-azo compounds rotated by 90° each, which confirms their birefringence. A morphologic study of the thin films deposited onto glass surfaces was performed, proving the good adhesion of both dyes. By thermal analysis and calorimetry, the melting temperatures were determined (~224–225 °C for both of them), as well as their decomposition pathways and thermal effects (enthalpy variations during undergoing processes); thus, good thermal stability was exhibited. The interaction of the two compounds with collagen in the suede was studied, as well as their antioxidant activity, advocating for good chemical stability and potential to be safely used as coloring agents in the food industry.

## 1. Introduction

Today, the colouring materials such as dyes and pigments are often synthesised and widely investigated for their useful assets and applications in various industrial sectors [1,2,3]. Dyes are usually organic compounds of natural origin or synthetic, having affinity to the substrate onto which they are applied [1,2,3]. Among dyes, the azoic compounds [2] are a well-known class of substances, which are frequently employed in specific applications for their physical and biological properties [4,5,6,7,8,9]: as liquid crystals [10,11,12,13,14,15,16,17,18,19,20,21,22,23,24,25,26,27,28], industrial [29,30,31,32,33,34,35,36,37,38,39] and food colouring agents [40,41,42,43,44,45], pharmaceuticals [35,36,46,47,48,49,50,51,52,53,54,55,56,57], etc. The molecules of some compounds contain only one chromophore azo-group (monoazo) [5,6,7,8,9,58], while others contain two (diazo) [59,60,61,62,63,64,65,66,67,68], four (tetrakisazo), or even more. The groups of atoms attached to the nitrogen are aromatic or heterocyclic systems [5,6,7,8,9,58]; the chemical structure of an azo dye is completed usually by the auxochrome groups (OH, OR, NH_2_, NHR, NR_2_).

The mains research on azo compounds refers to their reactivity and stability, as well as their antimicrobial activity. Stability is related to the photochemical [59], thermal [45,59,64,69,70] or enzymatic [59,69] decomposition of the azo compound. Yellow dyes such as tartrazine or sunset yellow have been employed extensively in the food-colouring sector [42], while new candidates are always welcomed; if they prove to have improved physical properties and accomplish safety standards, they may be further used for food preservation, preparation and consumption. However, if compared to natural dyes, the synthetic ones have to be extensively investigated in order to limit their possible harmful impact on human health and the environment, thus translating into higher vending costs [2].

In this paper, we report the synthesis of two bis-azo compounds (yellow dyes with two azodiphenylether groups) of anthracene: (i) BPA which stands for the 9,10-bis(((4′-phenyldiazenyl)-[1,1′-biphenyl]-4-yl)oxy)methyl)anthracene and (ii) BTA which stands for the 9,10-bis(((4′-p-tolyldiazenyl)-[1,1′-biphenyl]-4-yl)oxy) methyl)anthracene. Azoderivatives which contain two azo groups and two etheric groups are named bisazo-disethers and were synthesized by condensation of some azophenols with aromatic bis-chlorometilated derivatives in a basic medium. Here, the thermal, optical, spectral, adhesive and biological properties of the two bis-azo yellow dyes with two azodiphenylether groups of anthracene were investigated.

## 2. Experimental

### 2.1. Preparation of Bis-Azo Anthracene Compounds

**Figure 1:**
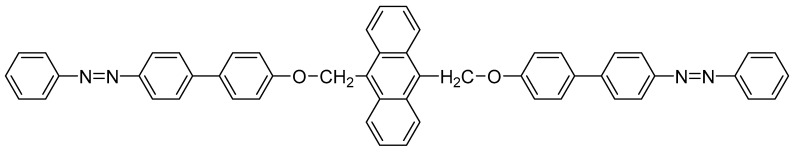
The structural formula of BPA.

**Figure 2:**
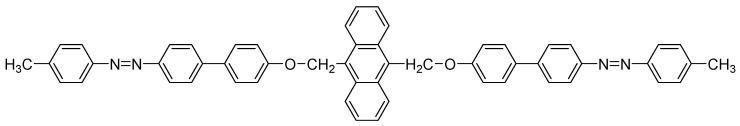
The structural formula of BTA.

**Figure 3:**
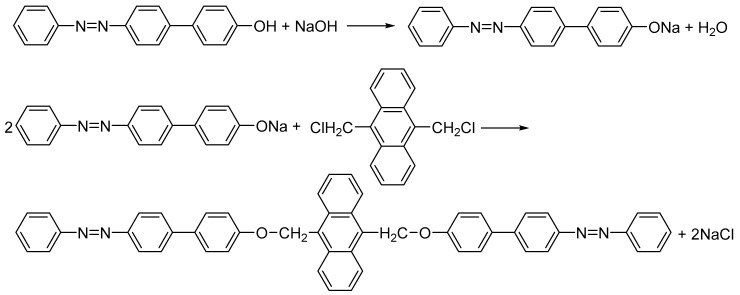
Obtaining scheme of BPA.

**Figure 4:**
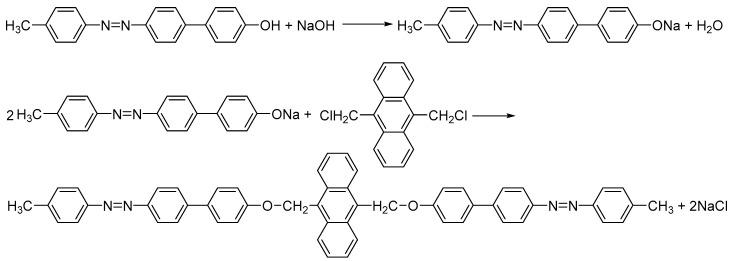
Obtaining scheme of BTA.

The preparation of bis-azo compounds is generally performed by condensing in a basic medium the bis-chloromethylated derivatives with the azophenolshas; the synthesis procedure was comprehensively described in several previous papers [71,72,73]. Here, we study two bis-chloromethylated compounds of anthracene, which were obtained by condensing the 9,10-bis(chloromethyl)anthracene with the 4-hydroxy-4′-phenylazo-diphenyl or with the 4-hydroxy-4′-(4-methyl-phenylazo)-diphenyl. Thus, the anhydrous sodium salts were obtained by reacting 4-hydroxy-4′-phenylazo-diphenyl or 4-hydroxy-4′-(4-methyl-phenylazo)-diphenyl with sodium hydroxide in an ethanol-benzene medium (in equal volumes). Further, the nucleophilic attack of the phenoxide anion on the biscloromethyl derivative of anthracene led to the formation of the bis-azo bis-eter. The precipitate obtained is separated on a G4 sintered glass filter, washed several times with distilled water and ethanol, and then dried in an oven at 105 °C [73]. The reaction product is purified by recrystallization from toluene; the compounds obtained are: the 9,10-bis(((4′-phenyldiazenyl)-[1,1′-biphenyl]-4-yl)oxy)methyl)anthracene (BPA), represented in Figure 1 and the 9,10-bis(((4′-p-tolyldiazenyl)-[1,1′-biphenyl]-4-yl)oxy)methyl)anthracene (BTA), represented in Figure 2. The sequences of reactions for obtaining BPA and BTA are shown in Figure 3 (BPA) and, respectively, Figure 4 (BTA).

As an identification mark, the two bis-azo compounds of anthracene have the melting temperatures of 224.4 °C (BPA) and 225.3 °C (BTA), and the molar masses of 750 g mol^−1^ (BPA) and 778 g mol^−1^ (BTA).

### 2.2. Methods and Techniques

By irradiating with electromagnetic radiation, the molecules of organic substances can store energy of translation, rotation, atomic vibration and electronic transition [42,43,74]. A commonly used technique for determining the chemical structure and identifying functional groups of organic substances is infrared absorption spectrometry [74,75,76,77,78,79,80,81,82,83,84,85].

The absorption of energy quanta, in the IR range (with wavelengths between 2.5–25 μm, or wavenumbers between 4000–400 cm^−1^), produces vibrations of atoms in organic molecules and rotations of groups of atoms in molecules or whole molecules [74,86]. In a nonlinear molecule, with n atoms, there are (3n − 6) normal vibrations of the atoms around their equilibrium positions. In a linear molecule, also with n atoms, there are (3n − 5) normal vibrations. Normal vibration (or normal vibration mode) occurs when all nuclei vibrate at the same frequency, in phase with each other [86].

Each polyatomic molecule has a defined vibration frequency, and the inherent energies of the molecular vibrations are quantified. When electromagnetic radiation, which has the same frequency as the vibration of atoms in a chemical bond, comes in contact with the molecule, it can absorb the radiation. Absorption occurs only if the atoms vibrating in the bond cause an oscillating dipole moment to appear, which can interact with the intensity of the electric field of radiation.

Only those molecular vibrations for which the dipole moment variation occurs will absorb radiation in the IR range of the spectrum. The higher the number of active frequencies in the IR of an organic molecule, the more asymmetric the molecule is [74].

Vibration spectra are recorded which are formed by absorption bands due to the vibrations of the atoms in the molecular structure of the analyzed compounds. The absorption lines are characterized by the wavenumber or wavelength of the absorbed radiation, and are attributed to the functional groups of the molecule. Thus, by positioning the spectral line in the IR spectrum, molecular structural elements can be identified.

The absorption spectra of infrared radiation by the compounds BPA and BTA were performed with the Spectrum100 FTIR Spectrometer from PerkinElmer, on the range of wave numbers 4000–400 cm^−1^, having the accuracy class of 0.01–60.00 cm^−1^. The universal attenuation total reflectance (UATR) accessory was used in the measurements, at a resolution of 4 cm^−1^, mediating 10 scans and permanent CO_2_/H_2_O correction [14,42,43,58].

The UV–Vis spectra of BPA and BTA compounds were recorded with Ocean Optics S2000 UV–Vis Spectrophotometer using a tungsten-halogen lamp model LS-1 from Ocean Optics and an Ar^+^ laser radiation with the wavelength of 476.5 nm [87,88].

Laser electronic fluorescence spectroscopy identifies the possible transitions between the energy levels of the electrons of the chemical elements in an organic or inorganic chemical compound, highlighting the energies and electronic configurations of the levels between which the transitions occur [42,43]. Atomic fluorescence was obtained with an Ar^+^ INOVA 308C laser from Coherent. Excitation radiation has the power of 440 mW and was measured with a Max Field Top 2 power meter, set for excitation radiation with λ = 476.5 nm. The electronic fluorescence spectra, on the wavelength range 400–900 nm of the excitation radiation, were recorded with an optical fiber placed perpendicular to the excitation radiation beam. The optical fiber is connected to the computer-connected Ocean Optics S2000 spectrometer.

Birefringence is the optical phenomenon of double refraction of light through a transparent medium with ordered molecules, in which there are differences in the orientation of the electric field of radiation that depend on the refractive index of light through the transparent medium [89,90]. Transparent solids that have the same refractive index in all directions of the crystal lattice (on all optical axes) are called optically isotropic. Through an isotropic crystal, light passes unpolarized at the same speed and refracts at a constant angle, regardless of the direction of radiation propagation through the crystal. In other transparent materials, called optically anisotropics, light is refracted in two rays (ordinary ray and extraordinary ray), which are totally polarized in perpendicular planes, i.e., have different propagation velocities in two directions through the transparent medium (different refractive indices) [75]. Optical anisotropy (birefringence) can be natural (ice, crystallized calcium carbonate, crystallized uric acid) [91], or induced by mechanical, electrical or magnetic actions.

The quantitative evaluation of the birefringence (*B*) of a crystal is made with the relation:

*B* = |*n_e_* − *n_o_*| (1)

where *n_o_* is the refractive index of the ordinary radius, and *n_e_* is the refractive index of the extraordinary radius, which come from the division of the incident radius. The optical path difference, D, or the delay (the retardation), *Γ*, of one wave from the other, are related to each other and are calculated with the relation:

*Γ* = *h* |*n_e_* − *n_o_*| (2)

where *h* is the thickness of the birefringent crystal. The greater the anisotropy of sample is, the higher the retardation (*Γ*) is. When the ordinary and extraordinary rays come out of the optically anisotropic crystals, the intensities of the electric fields of the rays vibrate in the same plane, forming a right angle between them. Ordinary beam delayed from extraordinary beam interferes with it, producing maximum interference (pronounced brightness) and minimum interference (extinction).

At interference, some birefringent crystals show changes in the color spectrum when observed in polarized light by cross-polarizers and when the sample cantilever rotates [92,93].

When a phase difference (retardation) occurs between linearly polarized light rays passing through an anisotropic crystal, the following situations may occur: When the retardation is an integer wavelength, the rays are recombined with the same orientation as at the crystal entrance, and this wave will be blocked by the analyzer (extinction) [93]. When the retardation is equal to half a wavelength, the rays are recombined in a direction perpendicular to the initial direction of polarization, and these waves will be transmitted entirely by the analyzer. A delay value is always characterized by the same combination of wavelengths and therefore the same color (maximum brightness) [93]. Through the interference of the two rays an image is born, which after the difference of optical path of the interfering rays, forms minimums and maximums. These depend on the angle at which the birefringent crystals are illuminated.

Birefringence determinations were performed in air at room temperature (RT). Bis-azo compounds were examined after their introduction between the cross polarizers of the polarized light microscope [94,95]. Bis-azo compounds were deposited on a glass plate COVN-024-200 from Labbox (24 × 24 mm, thickness 0.13–0.16 mm), by crystallization, on slow drying at ambient temperature, of the bis-azo solutions of the compounds, of 1% concentration in acetone. The crystals of bis-azo compounds whose optical anisotropy was determined were deposited on glass slides for optical microscopy. The crystals were obtained by slow drying, on slides, of *c* = 1% solutions in acetone, of the compounds BPA and BTA.

The adhesion to surfaces, of the bis-azo compounds of anthracene, was investigated by atomic force microscopy (AFM), in the non-contact style, using the PARK XE-100 SPM device. The sample blades were placed on the cantilever with a length of 125 mm, at a constant force of 40 N m^−1^ and an oscillation frequency of 275–373 kHz. The AFM technique allows the visualization and measurement with high precision, at high resolution, of the topography of surfaces [96] and the realization of ultrafast scans of surfaces at low temperatures [96,97,98,99]. A feature of the XE-100 Park Systems atomic force microscope is the recording of the optical image of the sample surface for the scanning region. AFM allows the topographic study of the sample surface, through three-dimensional (3D) and two-dimensional (2D) images; the 3D evaluation of the surface also introduces the height of the deposit as a measurable parameter.

Thermal analysis measurements (thermogravimetry-TG, derived thermogravimetry-DTG, differential thermal analysis-DTA and differential scanning calorimetry-DSC) were performed with a horizontal DIAMOND TG/DTA device from PerkinElmer Instruments, in a dynamic air atmosphere (at a flow rate of 150 cm^3^ min^−1^) with a heating rate of 10 °C min^−1^, from room temperature (RT) up to 600 °C by using alumina crucibles.

The interaction of bis-azo non-chlorinated anthracene compounds with proteins from collagen from the skin, was studied, using the FTIR spectrum of natural skin samples, untreated or treated with solutions in acetone of bis-azo non-chlorinated anthracene compounds, with a concentration of 2%. The Bruker ATR ZnSe FTIR spectrometer was used. The antioxidant activity of the bis-azo compounds of anthracene was also investigated; solutions of the bis-azo compounds were prepared in ethanol, with a concentration of 0.1%, when the dissolution is complete. Under these conditions, we evaluated the antioxidant activity of the bis-azo compounds of anthracene by the Folin–Ciocalteu (FC) spectrophotometric method [42]. A 200 μL of the sample was taken from the ethanol solutions of the compounds and placed in the test tube; to these solutions, 2.5 mL of FC reagent diluted 1:10 were added. After 4 min, 2 mL of 75 g L^−1^ sodium carbonate solution were added, then the mixtures were left for 2 h at room temperature.

## 3. Results and Discussions

### 3.1. FTIR Absorption Spectrum of Bis-Azo Compounds of Anthracene

**Figure 5:**
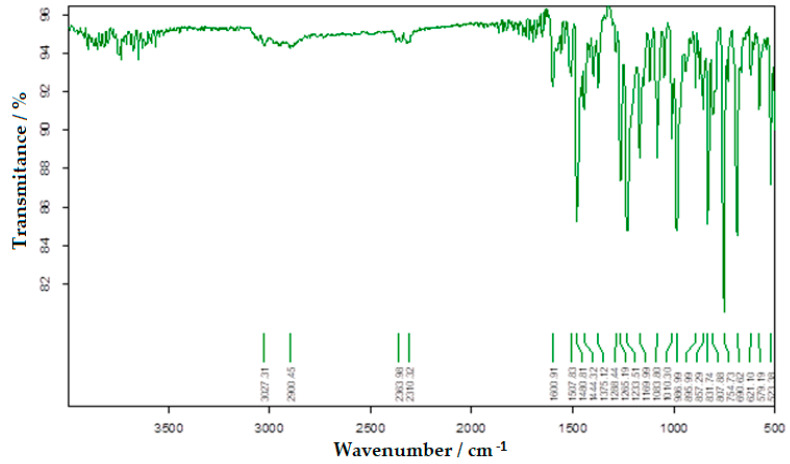
The FTIR spectrum of BPA.

**Figure 6:**
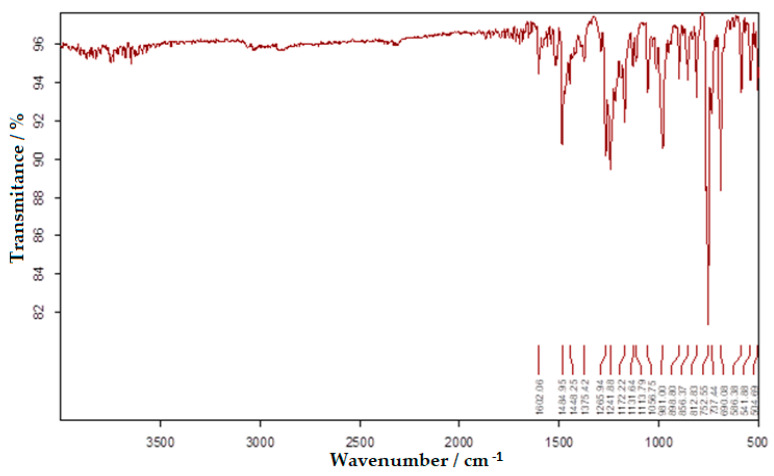
The FTIR spectrum of BTA.

The results of the IR analysis of bis-azo compounds, performed on the range of wave numbers 4000–500 cm^−1^, were represented in the transmittance mode, the absorption maxima (transmission minimums) of the infrared radiation being associated with the wavenumber values ($\bar{v}$), as in Figure 5 and Figure 6.

Azo compounds are quite difficult to identify by infrared spectroscopy, because the azo group absorbs in the same spectral range as the aromatic compounds, the *cis*-form having much more intense maxima than the trans-form. In Table 1 each value of the absorbed IR radiation is associated with the atomic bond or atomic group responsible for the absorption of the infrared photon.

The FTIR spectra of the two bis-azo compounds contain many similarities, due to the remarkable similarity between their chemical formulas. The only difference is the replacement in BPA of a hydrogen atom with a methyl group (in BTA). This causes the spectral lines of the radical -CH_3_ to appear in the IR spectrum of BTA, at 737, 542 and 505 cm^−1^. The most intense absorption bands of BPA and BTA, identified in IR spectrum were attributed to the following chemical bonds [58,73,74,86]:

- Absorption lines at 1601 or 1602 cm^−1^ are related to the conjugation of the azo group with aromatic nuclei.
- Absorption lines at 1481 or 1485 cm^−1^ are related to the vibration of the azo group.
- Absorption lines at 1234 or 1242 and 1170 or 1172 cm^−1^ are related to etheric vibrations.
- Absorption lines at 1375, 808 or 813 and 753 or 755 cm^−1^ are related to the anthracenic radical.

The FTIR spectroscopy confirms the molecular structure of the bis-azo compounds studied.

### 3.2. UV–Vis Absorption Spectrum of the Bis-Azo Compounds of Anthracene

Many organic compounds, especially azoic compounds, show characteristic infrared absorption, while in the visible range of the electromagnetic radiation spectrum, only colored substances show characteristic absorption. From the ultraviolet spectrum, they absorb only certain compounds, especially those aromatic or with conjugated systems [86].

**Figure 7:**
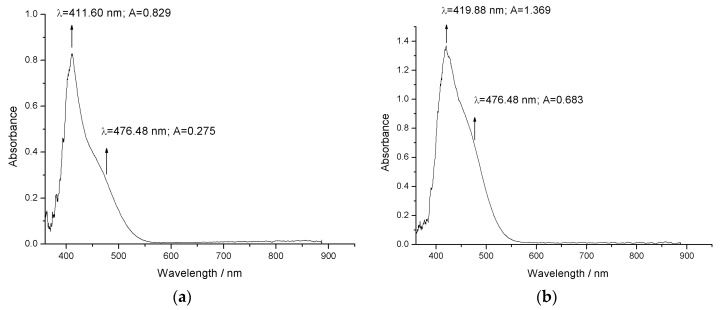
The UV–Vis spectrum of (**a**) BPA and (**b**) BTA.

The UV–Vis absorption spectra of bis-azo compounds are shown in Figure 7a (for BPA) and in Figure 7b (for BTA). Aqueous solutions were used, with a mass concentration *c* = 0.001%, of the two bis-azo compounds, in quartz cuvettes with a thickness of 10 mm. The water solubility of bis-azo compounds is very low, so the concentration cannot be increased too much. The spectrophotometer allows the elimination of the solvent contribution from the UV–Vis spectrum of the studied substance.

The UV–Vis absorption spectra of bis-azo compounds show that BPA has an absorption band with a maximum at 411.60 nm (with an absorbance of 0.829) and BTA has an absorption band with a maximum of 419.88 nm (with an absorbance of 1.369). It is observed that from the incident radiation, the two compounds also absorb the wavelength of 476.5 nm that can be emitted by the Ar^+^ laser. However, the absorbance is recorded at the wavelength of 476.48 nm, with *A* = 0.275 for BPA and *A* = 0.683 for BTA. This wavelength difference occurs because the wavelength of 476.48 nm is detected by one of the 2048 discrete detectors of the spectrometer, which has the value closest to the wavelength of 476.5 nm emitted by the Ar^+^ laser. Thus, the wavelength of 476.48 nm is adopted as the one for which the absorbances of the incident radiation will be considered.

From the experimental values, the ratio between the wavelength radiation absorbance of 476.48 nm and the wavelength radiation absorbance from the maximum UV–Vis of each bis-azo compound is calculated. These ratios are 0.33 for BPA and 0.50 for BTA. The values obtained from the reports indicate a quantum efficiency of the laser fluorescence for the wavelength of 476.5 nm, moderate for BPA and good for BTA.

### 3.3. The Electronic Laser Fluorescence of the Bis-Azo Compounds of Anthracene

Encouraged by the results of UV–Vis spectrophotometry, electronic fluorescence measurements were performed. Because bis-azo compounds are hardly soluble in water, solutions of compounds in acetone, with mass concentrations *c* = 1%, introduced in a quartz tank with a thickness of 10 mm were used to obtain the laser electronic fluorescence.

**Figure 8:**
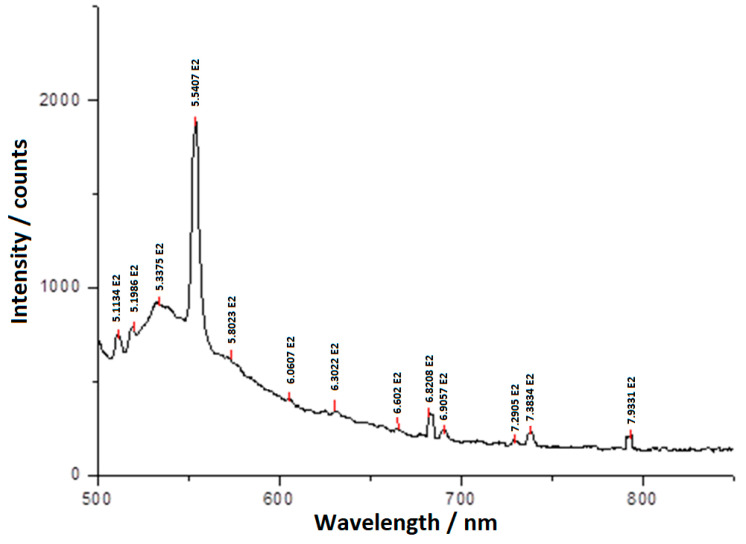
Laser electronic fluorescence spectrum of the BPA solution in acetone, with concentration *c* = 1%.

The laser electronic fluorescence spectrum of BPA, from Figure 8, contains the wavelengths of the de-excitation radiation, when the electron returns from the upper level (excited) to a lower level, with the emission of a photon. The interpretation of the laser electronic fluorescence spectrum was done using the NIST Atomic Database [89,90].

Table 2, Table 3, Table 4 and Table 5 contain the wavelengths of the quanta emitted at the de-excitation of each atom in the BPA composition, the calculated wavelengths, the electron energies at the upper and lower levels and the electronic configurations of the upper and lower electronic levels corresponding to these levels.

**Figure 9:**
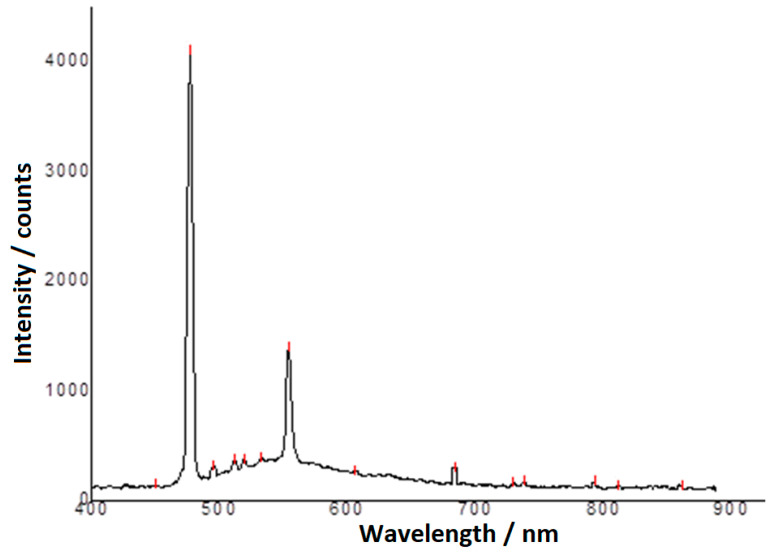
Laser electronic fluorescence spectrum of the BTA solution in acetone, with concentration *c* = 1%.

The laser electronic fluorescence spectrum of BTA, recorded on the same wavelength range of laser electronic fluorescence radiation (400–900 nm), is in Figure 9 and are systematized and presented in Table 6, Table 7, Table 8 and Table 9.

In Table 2, Table 3, Table 4, Table 5, Table 6, Table 7, Table 8 and Table 9 it is observed that some spectral lines correspond to two or three atoms (ions) of the studied substance, therefore they are more intense (477 nm emitted from carbon and nitrogen, 512 nm emitted from oxygen and nitrogen, 532–534 emitted from carbon, oxygen and nitrogen, 554 nm emitted from carbon and nitrogen, 684 nm emitted from carbon and oxygen, 738 nm emitted from oxygen and nitrogen). Other spectral lines (wavelengths or frequencies) come from a single atom (ion) of the investigated substance (450 nm emitted from nitrogen, 682 nm emitted from carbon, 690 and 729 nm emitted from oxygen). Some fluorescence quanta are identified in this paper for the first time in the laser fluorescence spectrum of some elements. We exemplify through the radiations having the wavelength of 534 nm emitted by carbon, oxygen and nitrogen, of 512, 520 and 630 nm emitted by oxygen and nitrogen, of 450, 477, 554, 665 and 738 nm emitted by nitrogen. Other identified laser fluorescence quanta confirm the previous results and coincide with the values in the NIST Atomic Database [89,90]. These spectral lines are attributed to the de-excitation transitions of the atoms of the two studied compounds.

### 3.4. Optical Anisotropy of the Bis-Azo Compounds

It has been established that anthracene bis-azo compounds studied in this research show optical anisotropy [100,101]. The birefringence images of BPA and BTA crystals were examined between the cross polarizers of the polarized light optical microscope [92,93,102].

During a complete rotation (360°) of the microscope holder on which the crystal sample is located, four positions (angles) of maximum illumination and four positions (angles) of extinction appear, which is explained by the birefringence property (on the phenomenon of double refraction).

**Figure 10:**
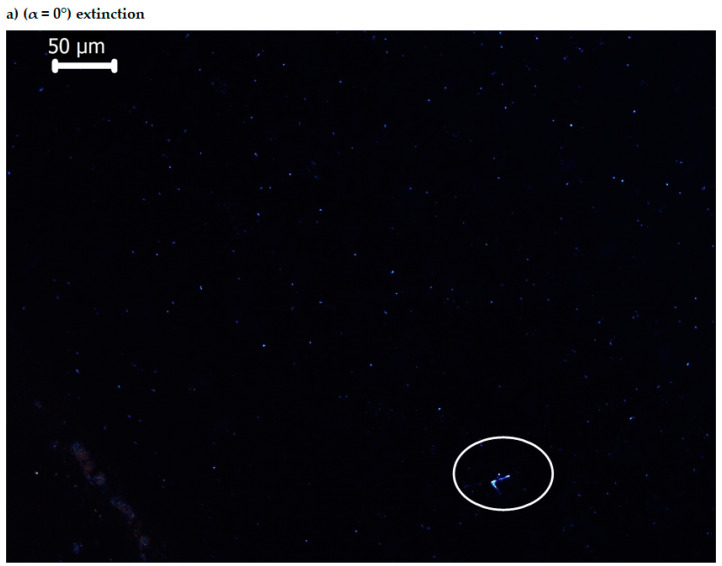

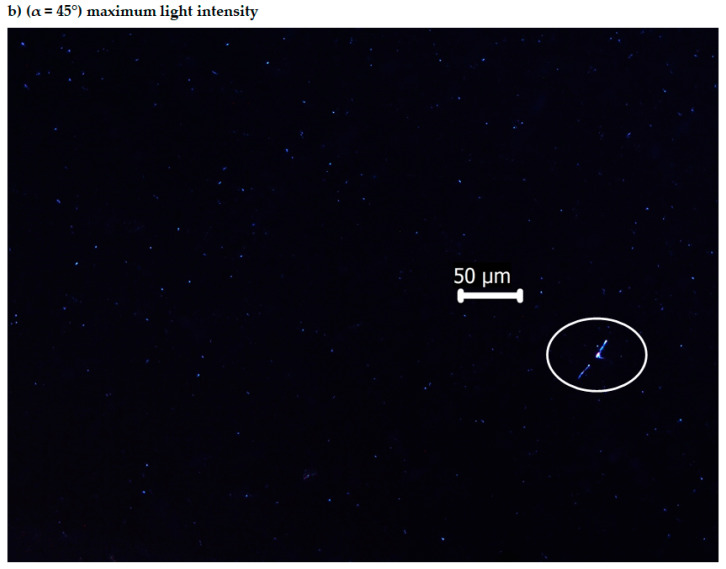
Optical images of BPA (*c* = 1%) with crossed polarizers.

**Figure 11:**
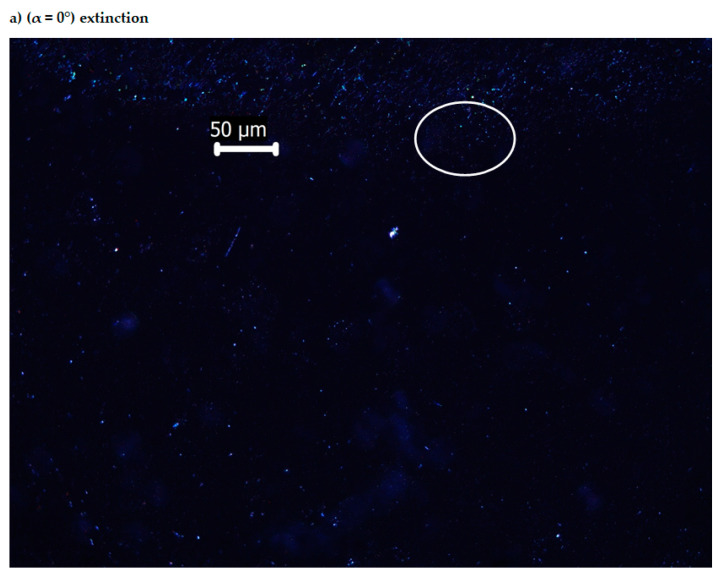

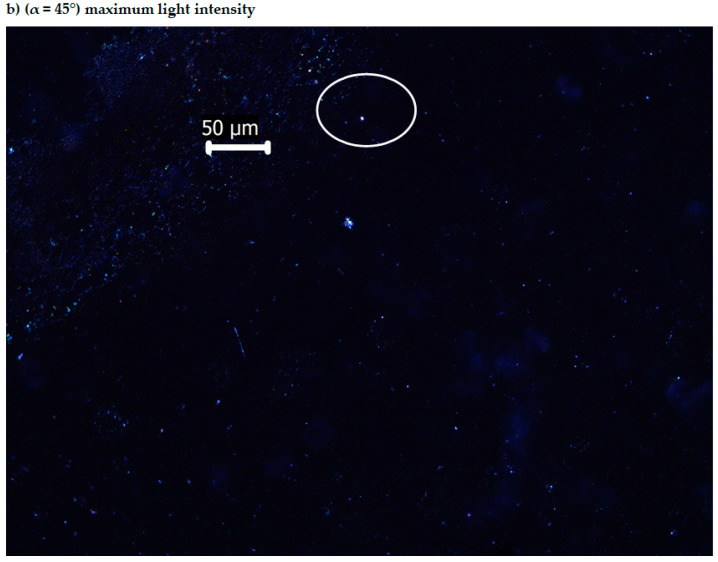
Optical images of BTA (*c* = 1%) with crossed polarizers.

Figure 10 presents only two images, which illustrate the birefringence of BPA crystallites, at positions of the table (lamella) of the optical microscope with polarized light for α = 0° (extinction) and α = 45° (maximum light intensity), the rest of the images being in the additional material. In Figure 11 are also represented only two images, which illustrate the birefringence of BTA crystallites, at positions of the table (lamella) of the optical microscope with polarized light for α = 0° (extinction) and α = 45° (maximum light intensity), the rest of the images being in the Supplementary Materials.

The images in Figure 10 and Figure 11 were obtained with a polarized light optical microscope, between crossed polarizers. The axis of the polarizing prism was oriented in the East-West direction, and the axis of the analyzing prism was oriented in the North–South direction. The axes are perpendicular to each other when the lamella is observed through the eyepieces. When the sample-free lamella is on the rotating table of the microscope, located between the cross polarizers, the result is a completely dark field [103].

Another dark field is observed if a section through the anisotropic crystals of the sample on the polarized light microscope slide is perpendicular to the optical axis. Then, the radius from the polarizer passes through that unchanged section, and when it reaches the analyzer it will not pass. In this case, the section through the anisotropic crystals is seen in black (extinction) [103]. The extinction will be repeated after a 90° rotation of the lamella (of the rotating table of the microscope), when the two refracted rays (ordinary and extraordinary rays) of crystal are parallel to the directions of vibration of light in the two nicols, and are eliminated by to the analyzer. If light passes from the polarizer to the analyzer, through the birefringent crystals, when the sample (microscope lamella) is rotated at angles other than the extinction angles (0, 90, 180, 270 and 360°), then a radius with a certain degree of brightness passes through the analyzer. The maximum brightness of the birefringent crystals is obtained when there is an angle of 45° between the polarizer and the analyzer, as seen in Figure 10 and Figure 11, images b—from the article—and d, f, h—from Supplementary Materials (45, 135, 225, 315°). Extinction appears in Figure 10 and Figure 11, images a—from the article—and c, e, g, i—from Supplementary Materials (0, 90, 180, 270, 360°).

The studied organic substances contain anthracene in their structure; the birefringence of anthracene was studied [103], establishing that the radiation absorbed by anthracene is strong in the visible region. The birefringence of the bis-azo compounds of anthracene, identified in this paper, is largely attributed to anthracene and may be a physical feature to be considered in medical applications.

### 3.5. Adhesion to the Surfaces of the Bis-Azo Compounds of Anthracene

This study refers to the adhesion of BPA and BTA compounds on a compact glass surface (microscope lamella). The sample for AFM was obtained by depositing, on the glass slide, three drops of anthracene bis-azo compound solution, with a concentration of 0.01% in acetone. After vibrating the lamella for two minutes, it is allowed to dry on the tray of an analytical balance at room temperature until the mass of the lamella remains constant.

**Figure 12:**
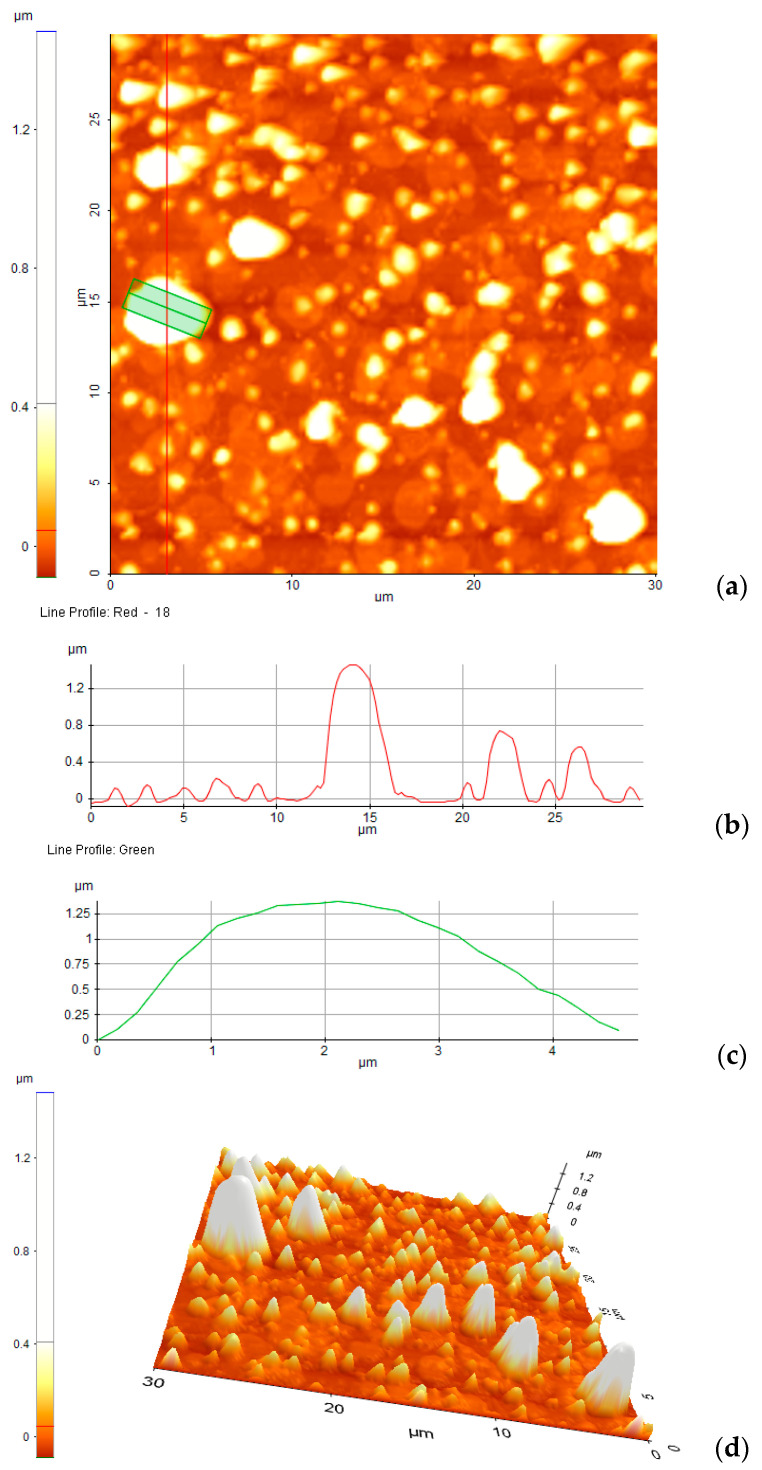
(**a**) The 2D topography of the surface on which BPA was deposited (30 × 30 μm); BPA deposition surface line profiles: (**b**) red and (**c**) green; (**d**) the 3D surface topography of the glass plate coated with BPA.

AFM measurements were performed at different magnifications [104]; some of them will be discussed in this paper. Figure 12a is a 2D representation of a surface with a size of 30 × 30 μm, on which BPA was deposited.

The 30 × 30 μm surface was scanned at a speed of 0.2 Hz, and the height of the BPA deposition is represented using the color code, shown to the left of Figure 12a. In Figure 12a are drawn two lines (a red line and a green line), whose profile is in Figure 12b,c.

Deposits are identified along the lines, in the form of hills, with different heights; the maximum height of the red line is 1453 nm and 1373 nm of the green line. The statistics of the quantities characteristic of the roughness, along the two lines, are in Table 10.

The meanings of the quantities in Table 10 are as follows: *Min* is the minimum height of the selected line profile; *Max* is the maximum height of the selected line profile; *Mid* is the average between the minimum and maximum height within the selected line. *Mid =* (*Min + Max*)/2; *Mean* is the average of the deposition heights on the entire selected line/area explored); *Rpv* (peak-to-valley) is the difference between maximum and minimum. *Rpv = Max* – *Min*; *Ra* is the roughness average; *Rq* is the root-mean-squared roughness. It is the standard deviation of the height value in the selected line.

(3) $$Rq=\sqrt{\bar{h^{2}}-{\left(\bar{h}\right)}^{2}}$$

where, $\bar{h^{2}}$ is the average of squares of height values in the selected line, and ${\left(\bar{h}\right)}^{2}$ is the square of the mean height of the heights in the line; *Rsk* is the skewness of the line; *Rku* is the kurtosis of the selected line. It indicates the “spikiness” of the sample surface along that line.

The 3D image of the surface with BPA deposition, from Figure 12d, more suggestively illustrates the 2D representation of Figure 12a, of the same area explored. Figure 12d shows the uniform dispersion of BPA crystals, with increasing surface roughness, due to the size of the crystals from tens of nanometers to a few micrometers.

**Figure 13:**
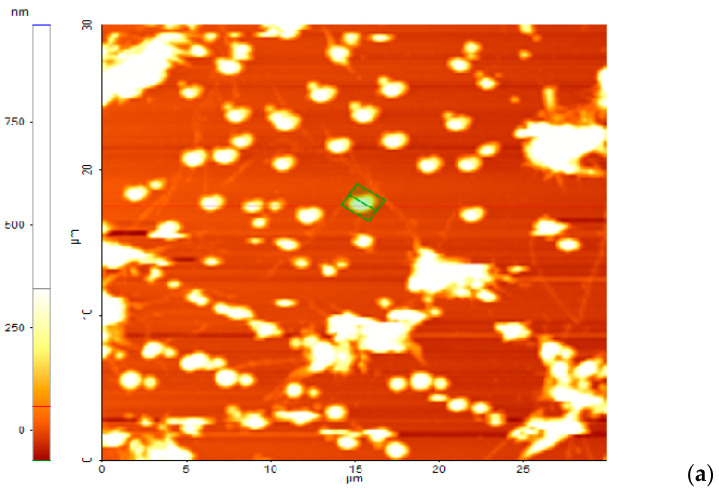

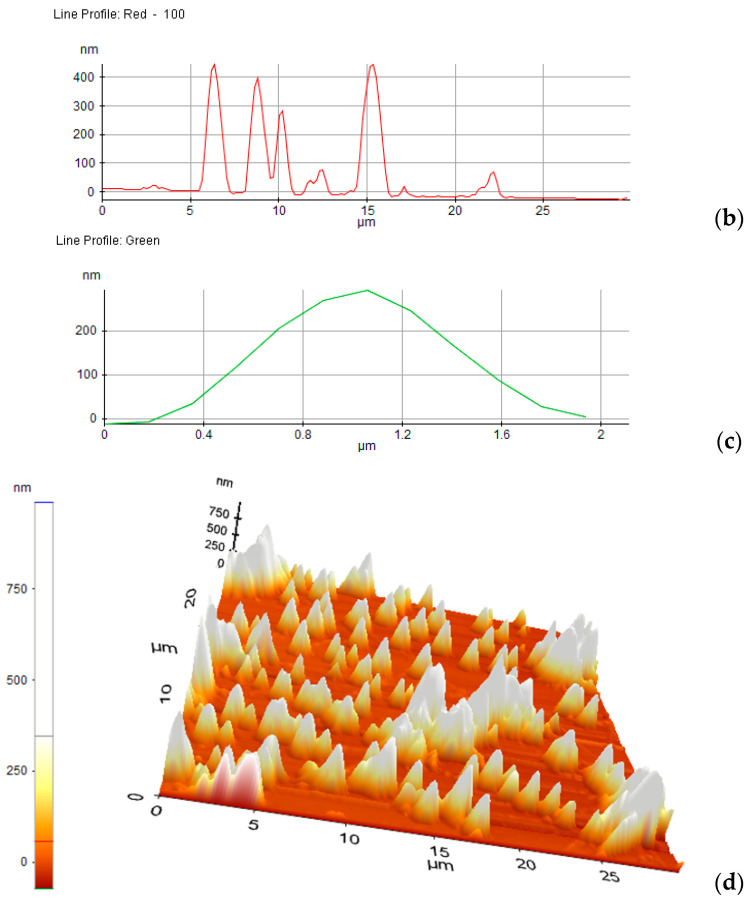
(**a**) 2D topography of the surface on which BTA was deposited (30 × 30 μm); BTA deposition surface line profiles: (**b**) red and (**c**) green; (**d**) the 3D surface topography of the glass plate coated with BTA.

The results of the AFM measurements performed on the BTA coated glass lamella, on the same surface with the size 30 × 30 μm, are presented in Figure 13a–d and in Table 11.

In this case, the crystals are well-formed, smaller than BPA and evenly dispersed, with the roughness (*Ra*) of ~100 nm, and with the skewness (*Rsk*) and the kurtosis (*Rku*) of the selected line, quite small. However, on the surface, small amounts of amorphous BTA are also observed.

The results of the statistical calculations of the quantities characteristic of the roughness, for the surfaces covered with BPA and BTA, are contained in Table 12.

### 3.6. Thermal Behaviour of BPA and BTA

**Figure 14:**
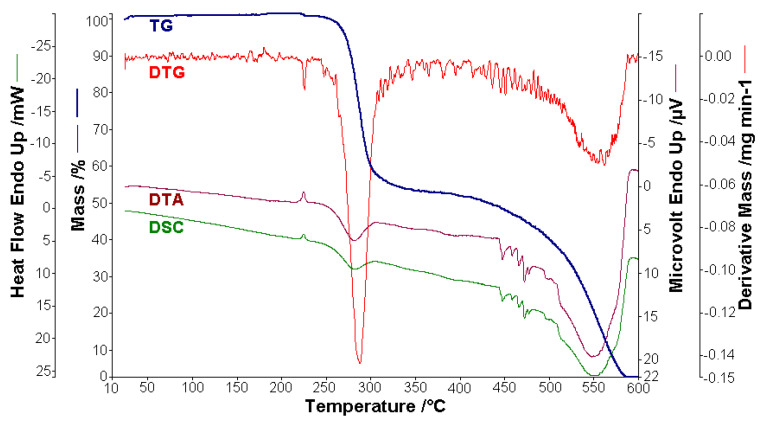
The thermoanalytical curves of BPA in a dynamic air atmosphere at 10 °C min^−1.^

**Figure 15:**
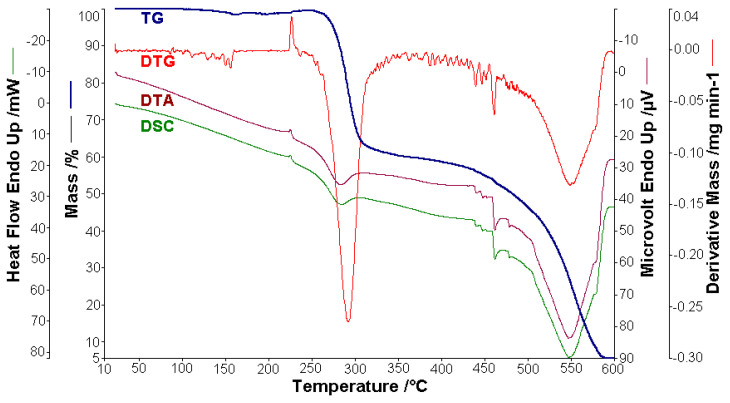
The thermoanalytical curves of BTA in a dynamic air atmosphere at 10 °C min^−1.^

Thermal analysis is often used to study materials, especially organic compounds and their composites, a category to which azoic-dye compounds belong [2,59,105,106,107,108,109,110,111,112]. This time we performed a detailed study of the thermal behavior of BPA and BTA compounds, in the temperature range RT–600 °C. The samples of BPA (0.778 mg) and BTA (1.858 mg) were heated in a dynamic air atmosphere (150 cm^−1^ min^−1^) with a heating rate of 10 °C min^−1^. The thermoanalytical curves of BPA (TG, DTG, DTA and DSC) are shown in Figure 14, and of BTA, in Figure 15.

**Figure 16:**
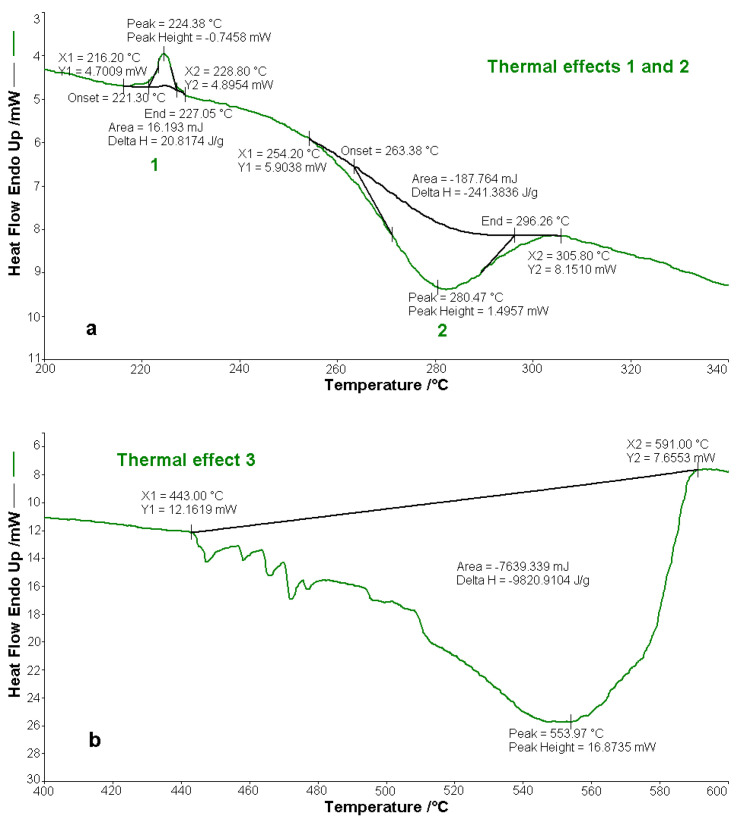
Thermal effects of decomposition of BPA in air: (**a**) thermal effect of melting (1) and thermal effect of the first decomposition (2), (**b**) thermal effect of the second decomposition (3).

**Figure 17:**
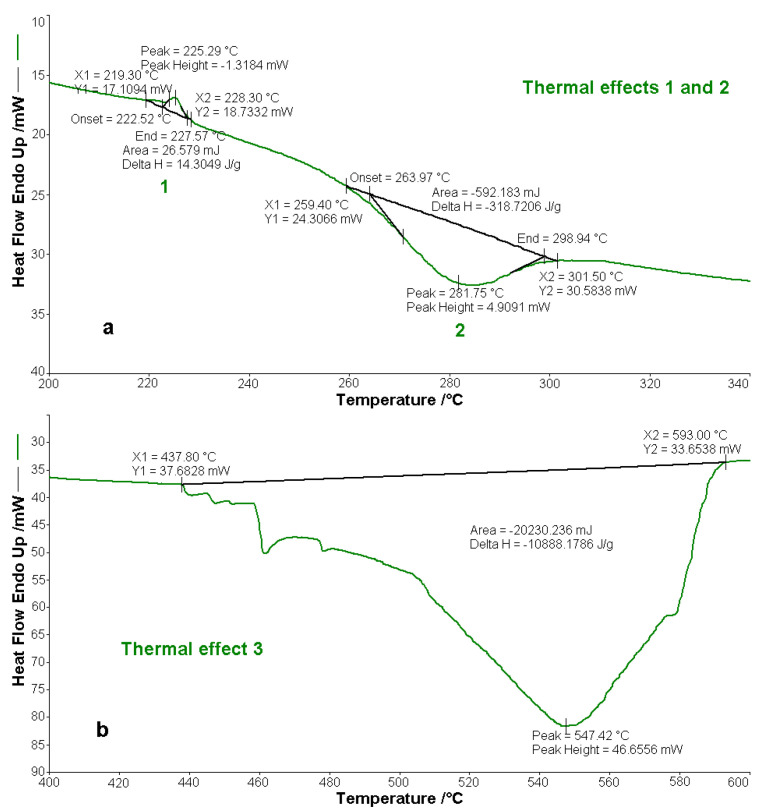
Thermal effects of decomposition of BTA in air: (**a**) thermal effect of melting (1) and thermal effect of the first decomposition (2), (**b**) thermal effect of the second decomposition (3).

The compounds BPA and BTA are thermally stable up to the temperature of 250 °C, after this temperature starting their oxidative decomposition. BPA melts at 224.4 °C, and BTA melts at 225.3 °C, absorbing the amounts of heat ∆*H*_0_, of 20.8 J g^−1^ (BPA) and 14.3 J g^−1^ (BTA). The decomposition of the two bis-azo compounds of anthracene occurs in two main stages. In the first decomposition stage, between temperatures of 250 and 366 °C BPA loses 47.5% of the mass, and between temperatures of 250 and 352 °C BTA loses 39.4% of the mass. The exothermic processes from the first stage are materialized in the decomposition enthalpies ∆*H*_1_, with the values of −241.4 J g^−1^ (BPA) and −318.7 J g^−1^ (BTA). In the second decomposition stage, BPA loses a mass of 50 % and BTA loses a mass of 54 %, the mass losses being accompanied by exothermic effects, with the enthalpy variations of ∆*H*_2_ of −9820.9 J g^−1^ (BPA) and −10,888.2 J g^−1^ (BTA). The thermal effects highlighted by the DSC curves of the thermal analysis are found in Figure 16 for BPA, and in Figure 17 for BTA.

Table 13 summarizes the numerical results of the thermal analysis and the identification of BPA decomposition products, and Table 14 the same data for BTA decomposition.

The experimental results, from Figure 16 and Figure 17 and from Table 13 and Table 14, regarding the thermal behavior of the two bis-azo compounds of anthracene, show that they melt at close temperatures (~225 °C), lose in the first stage of oxidative decomposition, in air, two benzene nuclei each, and in the second (multiple) stages they lose the rest of the molecule fragments. From BTA remains at 600 °C a 5 % residue, consisting of three carbon atoms.

### 3.7. The Biological Properties of Bis-Azo Non-Chlorinated Anthracene Compounds

#### 3.7.1. The Interaction of BPA and BTA with Proteins from Collagen

FTIR spectroscopy is a widely applied technique for the quantitative analysis of the secondary molecular structure of proteins. The proteins absorb in the infrared domain, having nine characteristic absorption bands, named: A, B, I–VII. The most important are the amide bands I (in the range of wave numbers 1700–1600 cm^−1^ and attributed to the stretching vibration of the C=O bond) and II (in the range of wave numbers 1600–1500 cm^−1^ and attributed to the vibration of stretching of the C–N bond and bending of the N–H bond). Amide band I characterizes the secondary structure of proteins as being more sensitive to structural changes than amide band II [42,110,111].

The absorption bands in the range of wave numbers 1450–1000 cm^−1^ belong to the deformation vibrations of the bonds in the −CH_3_ groups, of the tensile vibrations of the C–N bonds and the bending vibrations of the C–OH bonds of the skin proteins. In this field, the bis-azo anthracene compounds show poor absorption due to their low concentration [42,81,113].

**Figure 18:**
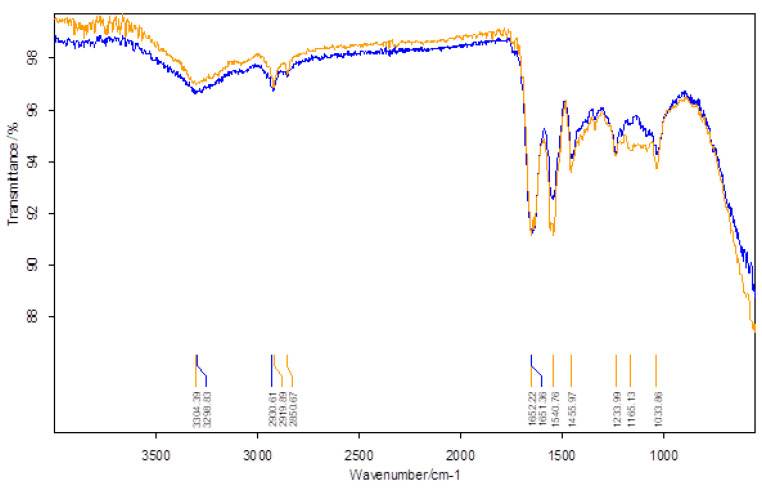
FTIR analysis: BPA untreated skin (blue) and BPA treated skin (brown).

**Figure 19:**
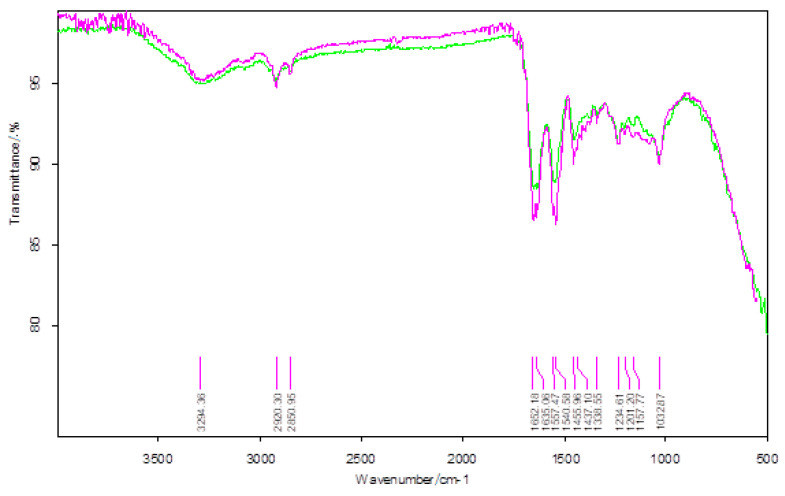
FTIR analysis: BTA untreated skin (purple) and BTA treated skin (green).

The FTIR analysis of a certain area on a suede was done first, then on that area was applied a solution of bis-azo unchlorinated anthracene compound (BPA or BTA), dissolved in acetone, with a concentration of 2 %. After drying the treated surface, for over a week, it was analyzed with the FTIR spectrometer. The results of the paired FTIR analyses: skin-skin with bis-azo anthracene compound deposition, are visualized in Figure 18 (BPA) and Figure 19 (BTA).

Table 15 and Table 16 retain the wavenumbers of radiation absorbed by the untreated suede skin and coated with a 2 % concentration solution of BPA (Table 15) and BTA (Table 16).

The specific frequencies of amide bands I are identified at 1651−1652 cm^−1^ in BPA and BPA and at 1634–1635 cm^−1^ in the case of BTA, depending on the local composition of the skin. The specific frequencies of the amide bands II are identified at values of wavenumbers of 1541–1556 cm^−1^ for both BPA and BTA deposition. The absorption bands of 1449–1456 cm^−1^, 1233–1236 cm^−1^ and 1032–1034 cm^−1^ can be attributed to the deformation vibrations of the -CH_3_ groups, the tensile vibrations of the C–N bonds and the bending of the bonds C–OH of skin proteins. There are also absorption frequencies characteristic of skin proteins: 2851, 1437, 1201 and 1158 cm^−1^, which are blocked by BTA. The presence of BPA and BTA on the skin surface is signaled by infrared absorption from 1163 cm^−1^, respectively from 1375 cm^−1^.

Collagen, cell and tissue proteins have a structure of FTIR absorption spectra, different from metabolic and structural proteins. Protein denaturation is influenced by hydrogen bonds in proteins and leads to changes in spectra. Molecular architecture and deformation of skin collagen proteins depend on the type of hydrogen bonds, and the interaction of collagen with bis-azo compounds of anthracene is reflected in the FTIR spectra of untreated skin and treated with bis-azo compounds of anthracene [42,113]. In the case analyzed by us, the interaction is weak, and the bis-azo compounds of anthracene can be washed off the skin.

#### 3.7.2. The Antioxidant Activity of BPA and BTA

The antioxidant activity of some substances is essential in preventing degenerative aging processes, such as Parkinson’s and Alzheimer’s diseases. Since the studied compounds (non-chlorinated bis-azo compounds of anthracene) do not possess potentially hydrogen-donating groups (such as hydroxyl/phenolic groups), it may be assumed that the O and N atoms can donate however electrons, i.e., have reducing properties (to be antioxidants). Previous research mentions substances that do not have phenolic groups and yet have antioxidant capabilities; those compounds contain carbamate groups and amides or amines in their molecule [114,115].

An important impediment in the study of the antioxidant activity of these azo compounds is the insolubility in water and the low solubility in alcohols. The bis-azo compounds of anthracene are soluble in acetone, but it is inappropriate to study the antioxidant activity of the acetone solutions of the compounds, as any living organism can be adversely affected by the acetone solvent. That is why we have prepared solutions of bis-azo compounds in ethanol, with a concentration of 0.1%, when the dissolution is complete. The antioxidant activity of the bis-azo compounds of anthracene was evaluated by the Folin–Ciocalteu (FC) spectrophotometric method [42], following the methodology described in the mmethods and techniques section. However, the blue complexes that were to form (blue tungsten oxide and blue molybdenum oxide) did not appear, however, therefore an antioxidant activity of the non-chlorinated bis-azo compounds of anthracene was not possible to be detected.

## 4. Conclusions

Following the synthesis of two bis-azo compounds of anthracene (BPA and BTA), several of their physical, thermal and biological properties were studied, allowing to formulate some conclusions.

1. The compounds are thermally stable in air up to a temperature of 250 °C, but melt before decomposition at 224.4 °C (BPA) and 225.3 °C (BTA), respectively. Their oxidative decomposition occurs in two exothermic stages with the decomposition enthalpies of −241.4 J g^−1^ (BPA) and −318.7 J g^−1^ (BTA) in the first stage, respectively of −9820.9 J g^−1^ (BPA) and −10,888.2 J g^−1^ (BTA) in the second step.
2. The FTIR spectra of the two bis-azo compounds of anthracene are similar, because their molecules contain mainly the same chemical bonds. The IR spectrum of BTA additionally contains the spectral lines of the radical -CH_3_, at 737, 542 and 505 cm^−1^.
3. The UV–Vis absorption spectra allowed the evaluation of the quantum efficiency of the laser fluorescence at the 476.5 nm wavelength of the Ar^+^ laser, used to excite the fluorescence of the atoms in the molecules of the compounds. The quantum laser fluorescence efficiency of 0.33 is moderate for BPA and 0.50 is good for BTA.
4. BPA and BTA molecules absorb light quanta with the wavelength of 476.5 nm, and electronic fluorescence quanta are emitted by de-excitation. The wavelengths and radiation energies emitted at the transition between electronic energy levels were identified, specifying the atom (the ion) or atoms (the ions) involved.
5. It has been established that the BPA and BTA crystals are anisotropic, they produce double refraction of light, i.e., they are birefringent. When the table of the optical microscope with polarized light is rotated by 90°, by the interference of ordinary and extraordinary rays, maximum brightness arises, which alternates with minimum brightness (extinction).
6. BPA and BTA dyes adhere well to glass surfaces, forming films with roughnesses of ~100 nm. The dispersion of the crystals on the surface, determined by AFM, is good. The dimensions of the crystals ranged from tens of nanometers to a few micrometers.
7. The interaction of BPA and BTA with collagen (suede) proteins is not very high, advocating for these compounds to be used safely as coloring agents; however, no significant antioxidant activity of these bis-azo anthracene compounds was identified.

## Figures and Tables

**Table 1:** Transmittance in the main infrared absorption maxima of BPA and BTA and their assignments [58,73,74,86].

BPA $\bar{v}$ (intensity *)/cm^−1^	BTA $\bar{v}$ (intensity *)/cm^−1^	Assignments
1600.91 (m)	1602.06 (m)	aromatic nucleus conjugated with the azo group; -N=N-;
1480.81 (s)	1484.95 (s)	-N=N-; aromatic ring stretching vibration;
1375.12 (m)	1375.42 (m)	polynuclear aromatic compounds;
1233.51 (s)	1241.88 (s)	C_Ar_-O-CH_2_- (antisymmetrical vibrations)
1169.99 (s)	1172.22 (s)	C_Ar_-O-CH_2_- (symmetrical vibrations)
807.88 (m)	812.83 (m)	anthracene radical;
754.73 (vs)	752.55 (vs)	anthracene radical;

* vs-very strong, s-strong, m-medium, w-weak.

**Table 2:** Atomic carbon fluorescence (BPA in acetone solution with *c* = 1%).

Ion	Wavelength Measured (in Air) *λ*/nm ±0.31 nm	Wavelength from the Literature (in Air) *λ*/nm	Wavelength Calculated Ritz (in Air) *λ*/nm	Energy of the Superior Level *E_k_*/eV	Energy of the Inferior Level *E_i_*/eV	The Electronic Configuration of the Superior Level	The Electronic Configuration of the Inferior Level
C I	476.84	476.6669	476.6667	81,325.81077	60,352.6584	2s^2^2p4p	2s^2^2p3s
C I		477.0023	477.0023	81,311.0544	60,352.6584	2s^2^2p4p	2s^2^2p3s
C II	533.75	-	533.4789	22.473901	20.150478	2s^2^6s	2s^2^4p
C III		-	533.7395	42.172212	39.849923	1s^2^2p(^2^P°)3d	1s^2^2s4d
C II	554.07	-	553.7609	21.732866	19.494540	2s^2^5p	2s^2^4s
C I		554.0756	554.0753	10.880080	8.64302361	2s^2^2p7s	2s^2^2p3p
C I		554.3817	554.3810	10.882983	8.64716033	2s^2^2p6d	2s^2^2p3p
C I	665.02	665.10	665.1008	10.7142897	8.85066275	2s^2^2p6s	2s^2^2p3p
C I	682.08	681.80	681.7976	88,638.99	73,975.92785	2s^2^2p(^2^P°_3/2_)7d	2s^2^2p3p

**Table 3:** Atomic nitrogen fluorescence (BPA in acetone solution with *c* = 1%).

Ion	Wavelength Measured (in Air) *λ*/nm ±0.31 nm	Wavelength from the Literature (in Air) *λ*/nm	Wavelength Calculated Ritz (in Air) *λ*/nm	Energy of the Superior Level *E_k_*/eV	Energy of the Inferior Level *E_i_*/eV	The Electronic Configuration of the Superior Level	The Electronic Configuration of the Inferior Level
N III	449.92	-	450.111	362,066	339,855.5	2s^2^7d	2s2p(^3^P°)3d
N IV	476.84	-	476.986	505,554.0	484,594.9	1s^2^2p(^2^P°)3d	1s^2^2p(^2^P°)3p
N II	511.54	-	511.4285	190,120.24	170 607.89	2s^2^2p3d	2s^2^2p3p
N I	519.99	-	5519.7902	19,233.177	0.000	2s^2^2p^3^	2s^2^2p^3^
N II		-	519.9501	243,012.77	223,785.51	2s2p^2^(^4^P)3d	2s2p^2^(^4^P)3p
N I		-	519.9837	112,807.567	93,581.550	2s^2^2p^2^(^3^P)5d	2s^2^2p^2^(^3^P)3p
N I		-	520.0257	19,224.464	0.000	2s^2^2p^3^	2s^2^2p^3^
N IV		-	520.041	484,594.9	465,371.0	1s^2^2p(^2^P°)3p	1s^2^2p(^2^P°)3s
N I		-	520.1606	112,801.031	93,581.550	2s^2^2p^2^(^3^P)5d	2s^2^2p^2^(^3^P)3p
N II	533.75	-	533.8729	244,353.31	225,627.47	2s2p^2^(^4^P)3d	2s2p^2^(^4^P)3p
N II		-	534.0207	244,391.88	225,671.22	2s2p^2^(^4^P)3d	2s2p^2^(^4^P)3p
N II	554.07	-	554.0061	223,643.30	205,597.97	2s2p^2^(^4^P)3p	2s2p^2^(^4^P)3s
N II		-	554.3471	223,688.45	205,654.22	2s2p^2^(^4^P)3p	2s2p^2^(^4^P)3s
N I	630.236	-	630.3915	112,609.612	96,750.840	2s^2^2p^2^(^3^P)6s	2s^2^2p^2^(^3^P)3p
N I	665.02	-	664.8256	112,807.567	97,770.180	2s^2^2p^2^(^3^P)5d	2s^2^2p^2^(^3^P)3p
N I		-	665.1147	112,801.031	97,770.180	2s^2^2p^2^(^3^P)5d	2s^2^2p^2^(^3^P)3p
N I	738.34	-	737.8513	110,299.974	96,750.840	2s^2^2p^2^(^3^P)4d	2s^2^2p^2^(^3^P)3p
N I		-	738.010	96,864.050	83,317.830	2s^2^2p^2^(^3^P)3p	2s^2^2p^2^(^3^P)3s

**Table 4:** Atomic oxygen fluorescence (BPA in acetone solution with *c* = 1%).

Ion	Wavelength Measured (in Air) *λ*/nm ±0.31 nm	Wavelength from the Literature (in Air) *λ*/nm	Wavelength Calculated Ritz (in Air) *λ*/nm	Energy of the Superior Level *E_k_*/eV	Energy of the Inferior Level *E_i_*/eV	The Electronic Configuration of the Superior Level	The Electronic Configuration of the Inferior Level
O V	511.54	-	511.4057	580,824.9	561,276.4	1s^2^2s3p	1s^2^2s3s
O III		-	511.775	394,197.9	374,663.52	2s2p^2^(^2^D)3s	2s2p^2^(^4^P)3p
O IV	519.68	-	519.822	573,696	554,464	2s2p(^3^P°)4s	2s2p(^1^P°)3p
O V	533.75	-	533.994	708,125.1	689,403.5	1s^2^2p(^2^P°_3/2_)3d	1s^2^2p(^2^P°_1/2_)3p
O I	630.24	-	630.0304	15,867.862	0.000	2s^2^2p^4^	2s^2^2p^4^
O II	665.02	665.2563	665.2559	246,455.629	231,427.970	2s^2^2p^2^(^3^P)4p	2s^2^2p^2^(^3^P)3d
O II	690.57	690.6443	690.6436	245,903.224	231,427.970	2s^2^2p^2^(^3^P)4p	2s^2^2p^2^(^3^P)3d
O V		-	690.727	704,182.0	689,708.5	1s^2^2p(^2^P°_1/2_)3d	1s^2^2p(^2^P°_3/2_)3p
O II		690.7872	690.7873	245,768.37	231,296.126	2s^2^2p^2^(^3^P)4p	2s^2^2p^2^(^3^P)3d
O II	729.05	728.7894	728.7900	246,320.086	232,602.492	2s^2^2p^2^(^3^P)4p	2s^2^2p^2^(^3^P)3d
O II		729.2129	729.2124	246,455.629	232,745.981	2s^2^2p^2^(^3^P)4p	2s^2^2p^2^(^3^P)3d
O II		729.2962	729.2965	246,455.629	232,747.562	2s^2^2p^2^(^3^P)4p	2s^2^2p^2^(^3^P)3d
O II	738.34	-	738.0307	246,291.822	232,745.981	2s^2^2p^2^(^3^P)4p	2s^2^2p^2^(^3^P)3d
O II		738.1118	738.1168	246,291.822	232,747.562	2s^2^2p^2^(^3^P)4p	2s^2^2p^2^(^3^P)3d
O II		738.3744	738.3758	266,588.33	253,048.82	2s^2^2p^2^(^1^D)4p	2s^2^2p^2^(^1^D)3d

**Table 5:** Systematization of spectral lines of electronic laser fluorescence of BPA atoms (ions).

The Measured Wavelength *λ*/nm (Intensity) *	The Atom (ion) in BPA That Has Electronic Fluorescence at a Certain Wavelength		
	C	O	N
449.92 (vw)	-	-	Yes
476.84 (vs)	Yes	-	Yes
511.54 (s)	-	Yes	Yes
519.99 (m)	-	Yes	Yes
533.75 (s)	Yes	Yes	Yes
554.07 (vs)	Yes	-	Yes
630.22 (w)	-	Yes	Yes
665.02 (w)	Yes	Yes	Yes
682.08 (s)	Yes	-	-
690.57 (m)	-	Yes	-
729.05 (w)	-	Yes	-
738.34 (m)	-	Yes	Yes

* vs-very strong, s-strong, m-medium, w-weak.

**Table 6:** Atomic carbon fluorescence (BTA in acetone solution with *c* = 1%).

Ion	Wavelength Measured (in Air) *λ*/nm ±0.31 nm	Wavelength from the Literature (in Air) *λ*/nm	Wavelength Calculated Ritz (in Air) *λ*/nm	Energy of the Superior Level *E_k_*/eV	Energy of the Inferior Level *E_i_*/eV	The Electronic Configuration of the Superior Level	The Electronic Configuration of the Inferior Level
C I	476.84	476.6669	476.6667	81,325.81077	60,352.6584	2s^2^2p4p	2s^2^2p3s
C I		477.0023	477.0023	81,311.0544	60,352.6584	2s^2^2p4p	2s^2^2p3s
C I	532.37	-	532.4065	87,633.77	68,856.35208	2s^2^2p6d	2s^2^2p3p
C II	554.07	-	553.7609	175,287.39	157,234.07	2s^2^5p	2s^2^4s
C I		554.0756	554.0753	87,753.763	69,710.68728	2s^2^2p7s	2s^2^2p3p
C I	684.37	681.80	681.7976	88,638.99	73,975.92785	2s^2^2p(^2^P°_3/2_)7d	2s^2^2p3p

**Table 7:** Atomic nitrogen fluorescence (BTA in acetone solution with *c* = 1%).

Ion	Wavelength Measured (in Air) *λ*/nm ±0.31 nm	Wavelength from the Literature (in Air) *λ*/nm	Wavelength CalculatedRitz (in Air) *λ*/nm	Energy of the Superior Level *E_k_*/eV	Energy of the Inferior Level *E_i_*/eV	The Electronic Configuration of the Superior Level	The Electronic Configuration of the Inferior Level
N III	449.92	-	450.111	362,066	339,855.5	2s^2^7d	2s2p(^3^P°)3d
N IV	476.84	-	476.986	505,554.0	484,594.9	1s^2^2p(^2^P°)3d	1s^2^2p(^2^P°)3p
N II	511.54	-	511.4285	190,120.24	170,572.61	2s^2^2p3d	2s^2^2p3p
N II	519.19	-	519.0380	243,046.56	223,785.51	2s2p^2^(^4^P)3d	2s2p^2^(^4^P)3p
N II		-	519.1965	242,986.76	223,731.59	2s2p^2^(^4^P)3d	2s2p^2^(^4^P)3p
N III	532.37	532.082	532.087	339,855.5	321,066.8	2s2p(^3^P°)3d	2s2p(^3^P°)3p
N II		-	532.0958	244,391.88	225,603.50	2s2p^2^(^4^P)3d	2s2p^2^(^4^P)3p
N II	554.07	-	554.0061	223,643.30	205,597.97	2s2p^2^(^4^P)3p	2s2p^2^(^4^P)3s
N II		-	554.3471	223,688.45	205,654.22	2s2p^2^(^4^P)3p	2s2p^2^(^4^P)3s
N I	738.02	-	737.8513	110,299.974	96,750.840	2s^2^2p^2^(^3^P)4d	2s^2^2p^2^(^3^P)3p
N I		-	738.010,	96,864.050	83,317.830	2s^2^2p^2^(^3^P)3p	2s^2^2p^2^(^3^P)3s

**Table 8:** Atomic oxygen fluorescence (BTA in acetone solution with *c* = 1%).

Ion	Wavelength Measured (in Air) *λ*/nm ±0.31 nm	Wavelength from the Literature (in Air) *λ*/nm	Wavelength Calculated Ritz (in Air) *λ*/nm	Energy of the Superior Level *E_k_*/eV	Energy of the Inferior Level *E_i_*/eV	The Electronic Configuration of the Superior Level	The Electronic Configuration of the Inferior Level
O V	511.54	-	511.4057	580,824.9	561,276.4	1s^2^2s3p	1s^2^2s3s
O III		-	511.775	394,197.9	374,663.52	2s2p^2^(^2^D)3s	2s2p^2^(^4^P)3p
O II	519.19	519.0496	519.0498	233,430.53	214,169.920	2s^2^2p^2^(^3^P)3d	2s^2^2p^2^(^3^P)3p
O II	532.37	532.2525	532.2502	267,298.23	248,515.30	2s^2^2p^2^(^3^P)6s	2s^2^2p^2^(^3^P)4p
O II	684.37	-	684.4098	245,903.224	231,296.126	2s^2^2p^2^(^3^P)4p	2s^2^2p^2^(^3^P)3d
O II	729.37	729.2129	729.2124	246,455.629	232,745.981	2s^2^2p^2^(^3^P)4p	2s^2^2p^2^(^3^P)3d
O II		729.2962	729.2965	246,455.629	232,747.562	2s^2^2p^2^(^3^P)4p	2s^2^2p^2^(^3^P)3d
O II		729.6310	729.6294	246,455.629	232,753.816	2s^2^2p^2^(^3^P)4p	2s^2^2p^2^(^3^P)3d
O II	738.02	-	738.0307	246,291.822	232,745.981	2s^2^2p^2^(^3^P)4p	2s^2^2p^2^(^3^P)3d
O II		738.1118	738.1168	246,291.822	232,747.562	2s^2^2p^2^(^3^P)4p	2s^2^2p^2^(^3^P)3d

**Table 9:** Systematization of spectral lines of electronic laser fluorescence of BTA atoms (ions).

The Measured Wavelength *λ*/nm (Intensity) *	The Atom (ion) in BTA That Has Electronic Fluorescence at a Certain Wavelength		
	C	O	N
449.92 (vw)	-	-	Yes
476.84 (vs)	Yes	-	Yes
511.54 (w)	-	Yes	Yes
519.19 (w)	-	Yes	Yes
532.37 (w)	Yes	Yes	Yes
554.07 (s)	Yes	-	Yes
684.37 (m)	Yes	Yes	-
729.37 (w)	-	Yes	-
738.02 (w)	-	Yes	Yes

* vs-very strong, s-strong, m-medium, w-weak, vw-very weak.

**Table 10:** Statistics of the quantities characteristic of the roughness on the lines on the BPA surface.

Line	*Min*/ nm	*Max*/ nm	*Mid*/ nm	*Mean*/ nm	*Rpv*/ nm	*Rq*/ nm	*Ra*/ nm	*Rsk*	*Rku*
Red	84	1453	684	216	1573	394	284	−1.979	5.910
Green	0	1373	687	836	1374	456	405	0.435	1.740

**Table 11:** Statistics of the quantities characteristic of the roughness on the lines on the BTA surface.

Line	*Min*/ nm	*Max*/ nm	*Mid*/ nm	*Mean*/ nm	*Rpv*/ nm	*Rq*/ nm	*Ra*/ nm	*Rsk*	*Rku*
Red	−29	445	208	40	474	113	77	−2.277	7.115
Green	−12	292	140	119	303	108	97	−0.270	1.552

**Table 12:** Statistics of the quantities characteristic of the roughness on the surfaces covered with BPA and BTA.

Sample	*Min*/ nm	*Max*/ nm	*Mid*/ nm	*Mean*/ nm	*Rpv*/ nm	*Rq*/ nm	*Ra*/ nm	*Rsk*	*Rku*
BPA	−88	1482	697	47	1570	186	107	−4.017	22.630
BTA	−73	988	458	57	1060	147	101	−2.531	9.760

**Table 13:** Thermal analysis of BPA.

Temperature Range/ °C	Mass Loss (Experimental)/ %	Mass Loss (Theoretical)/ %	Identified Lost Groups	ΔH/ J g^−1^	Observations
216.2–228.8	-	-	-	∆*H*_0_ = 20.8	endotherm-melting
254.2–305.8	47.48	48.00	2(C_6_H_4_-C_6_H_4_) 2N_2_	∆*H*_1_ = −241.4	the first exothermic oxidative decomposition
443.0–591.0	50.00	52.00	2C_6_H_5_ 2(CH_2_-O) C_14_H_8_	∆*H*_2_ = −9820.9	the second exothermic oxidative decomposition

**Table 14:** Thermal analysis of BTA.

Temperature Range/ °C	Mass Loss (Experimental)/ %	Mass Loss (Theoretical)/ %	Identified Lost Groups	ΔH/ J g^−1^	Observations
219.3–228.3	-	-	-	∆*H*_0_ = 14.4	endotherm-melting
259.4–301.5	39.36	39.07	2(C_6_H_4_-C_6_H_4_)	∆*H*_1_ = −318.7	the first exothermic oxidative decomposition
437.8–593.0	54.00	56.30	2N_2_ 2(CH_3_-C_6_H_4_) 2(CH_2_-O) C_11_H_8_	∆*H*_2_ = −10,888.2	the second exothermic oxidative decomposition
600	5.00	4.63	3C	-	residue

**Table 15:** Wavenumbers of infrared radiation absorption in the case of BPA.

Wavenumber/cm^−1^	
BPA-Untreated Skin	BPA-Treated Skin
3299	3304
2931	2920
-	2851
1651	1652
1549	1541
1454	1456
1236	1234
-	1163
1033	1034

**Table 16:** Wavenumbers of infrared radiation absorption in the case of BTA.

Wavenumber/cm^−1^	
BTA-Untreated Skin	BTA-Treated Skin
3294	3284
2920	2921
2851	-
1652	1651
1635	1634
1557	1549
1456	1449
1437	-
-	1375
1339	1337
1235	1233
1201	-
1158	-
1033	1032

## Supplementary Materials

The following are available online, Figure S1: Optical images of BPA (*c* = 1%) with crossed polarizers, Figure S2: Optical images of BTA (*c* = 1%) with crossed polarizers.
