# Supplementary material for: Physical, Thermal and Biological Properties of Yellow Dyes with Two Azodiphenylether Groups of Anthracene
Source: Molecules. 2020 Dec 6;25(23):5757. doi: 10.3390/molecules25235757 (PMC7731036; doi:10.3390/molecules25235757)
Supplement: Supplementary file 1 [file molecules-25-05757-s001.pdf]

Supplemnetary Figure 10 Optical images of BPA (c = 1%) with crossed polarizers

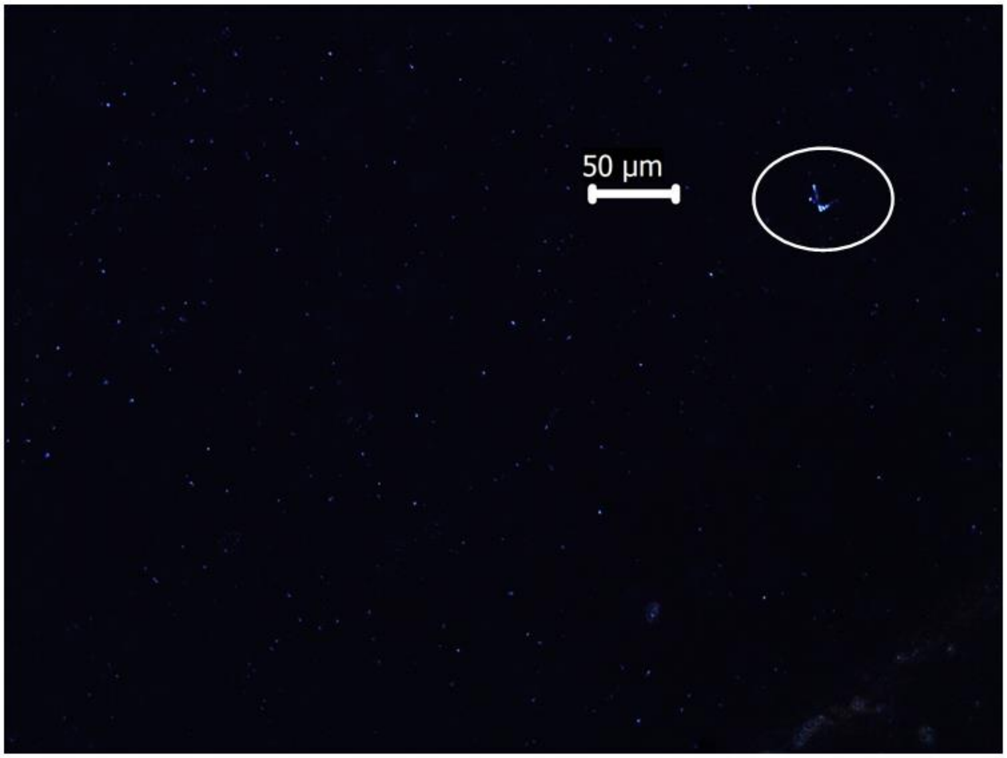

S10-c (α = 90°) extinction

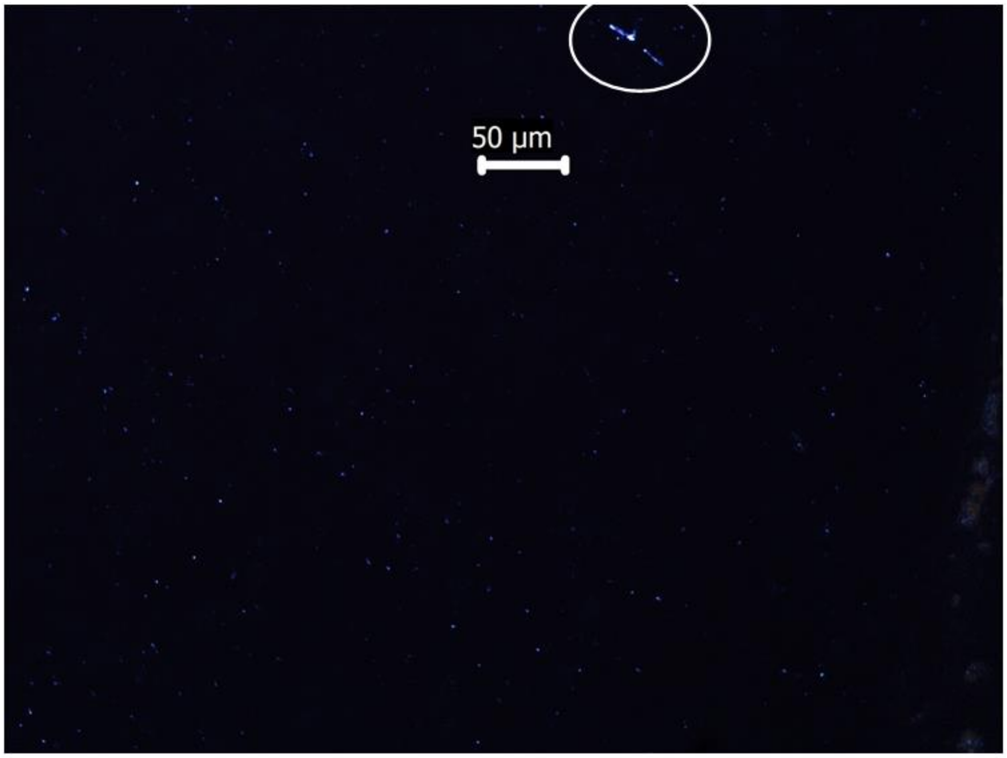

S10-d (α = 135°) maximum light intensity

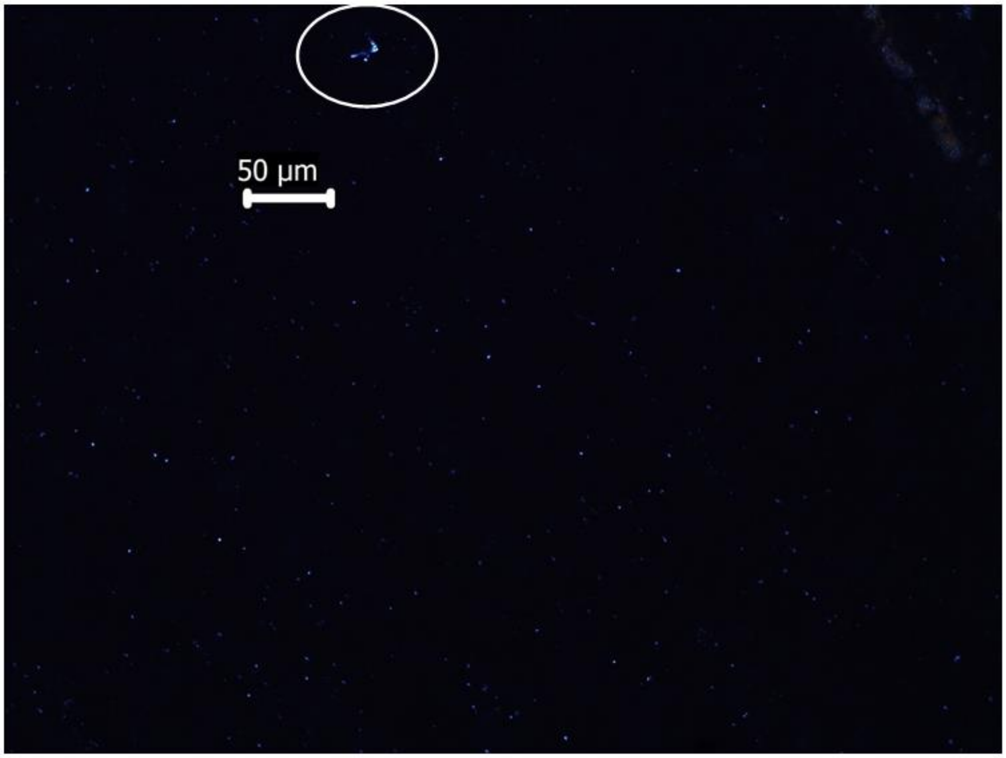

S10-e (α = 180°) extinction

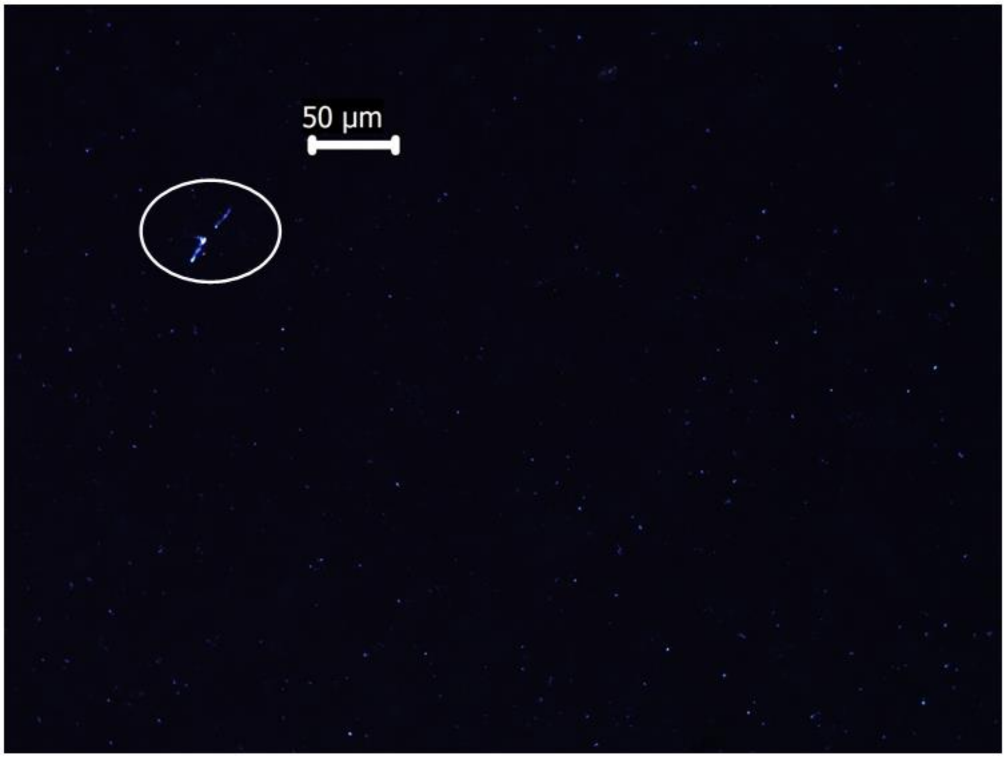

S10-f (α = 225°) maximum light intensity

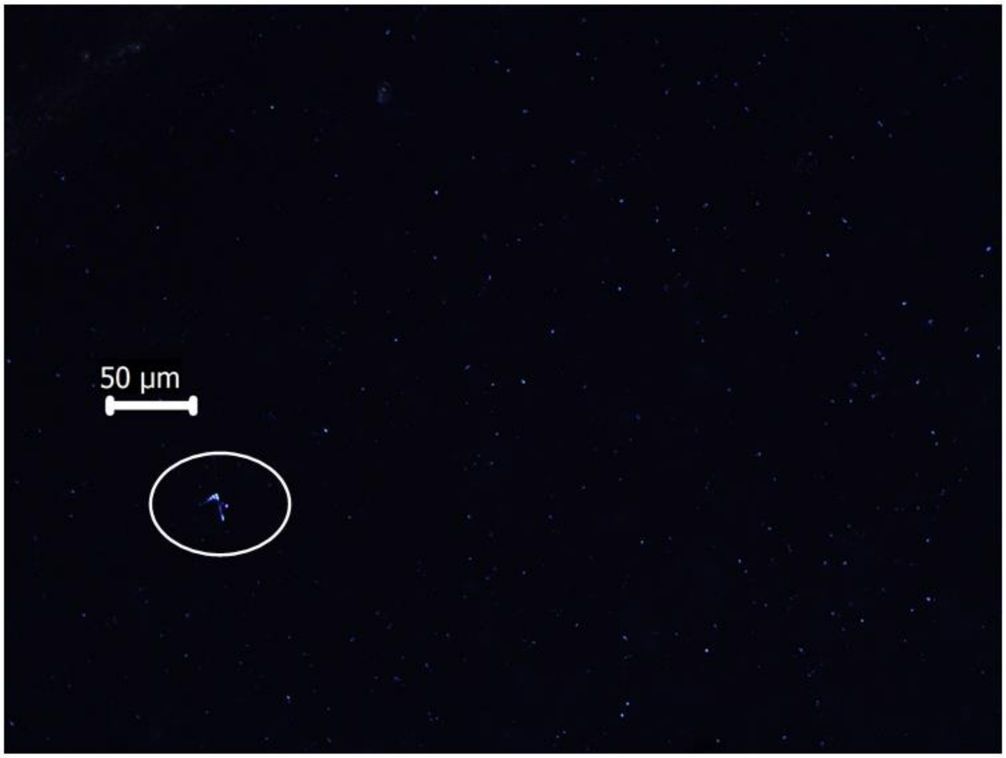

S10-g (α = 270°) extinction

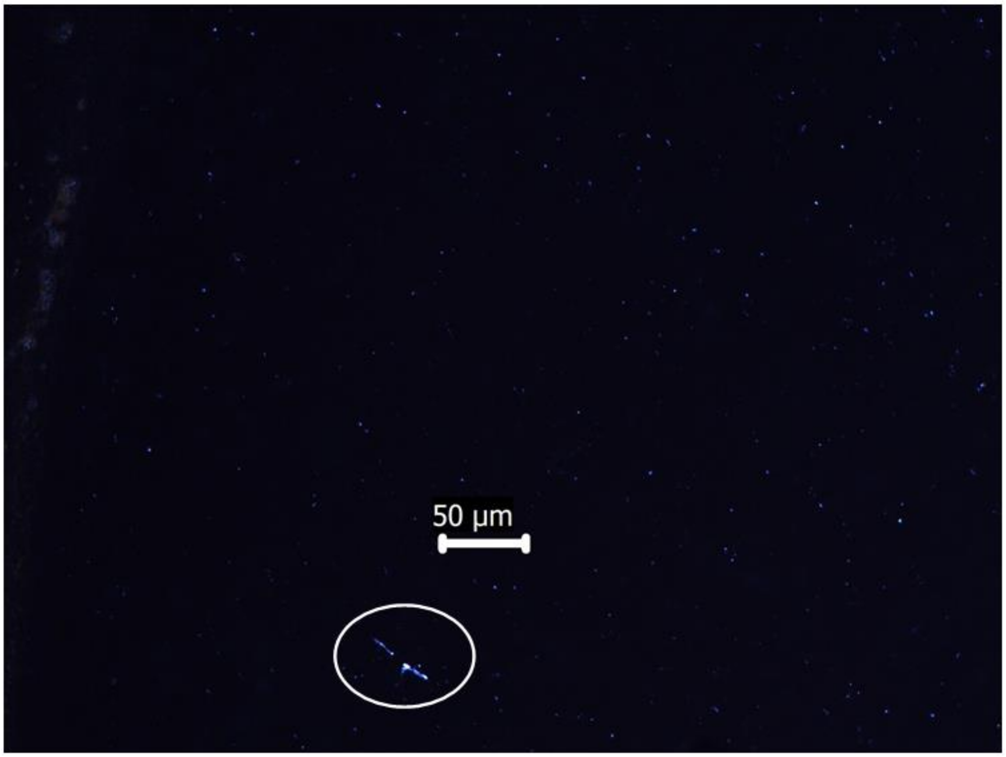

S10-h (α = 315°) maximum light intensity

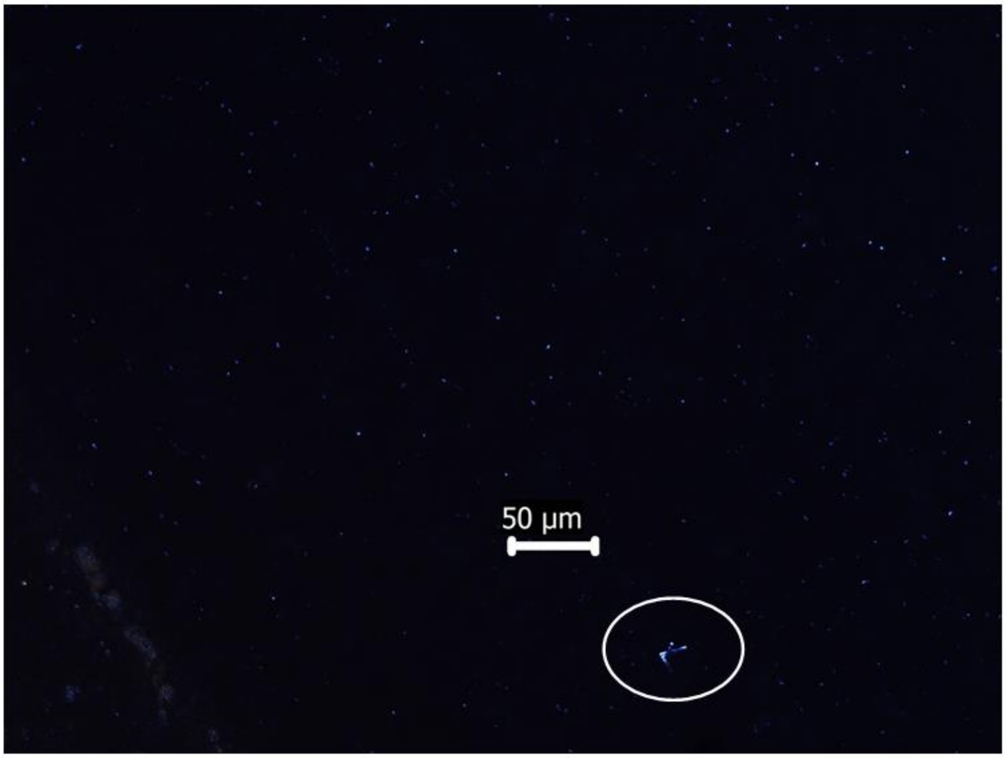

S10-i (α = 360°) extinction

Supplementary Figure 11 Optical images of BTA (c = 1%) with crossed polarizers

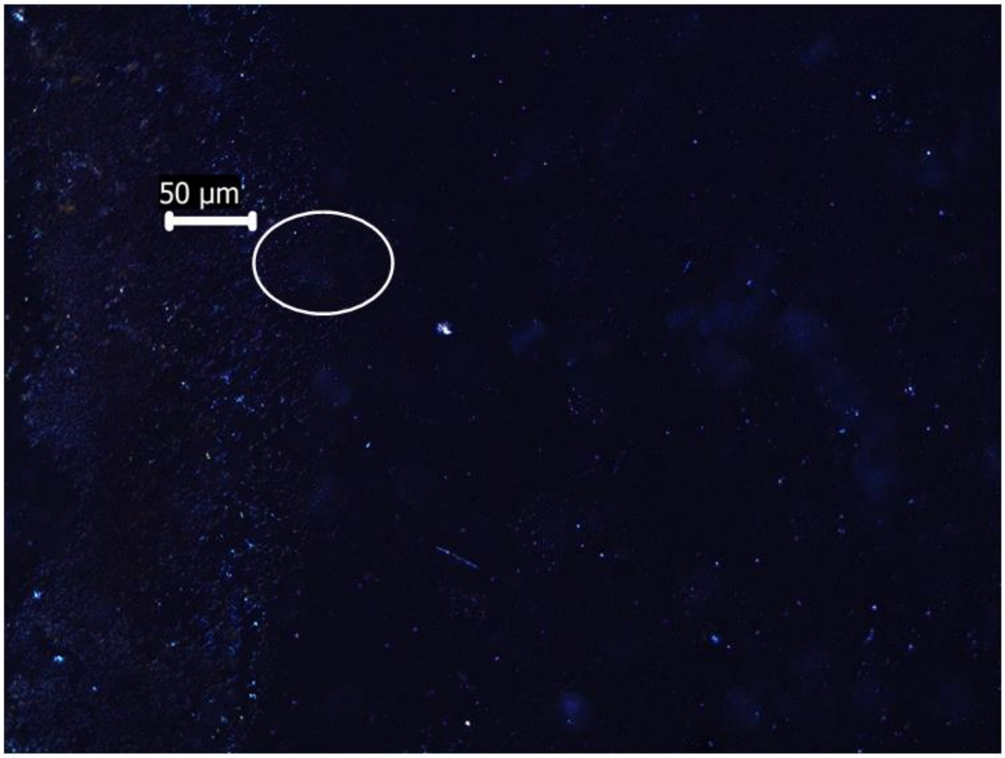

S11-c (α = 90°) extinction

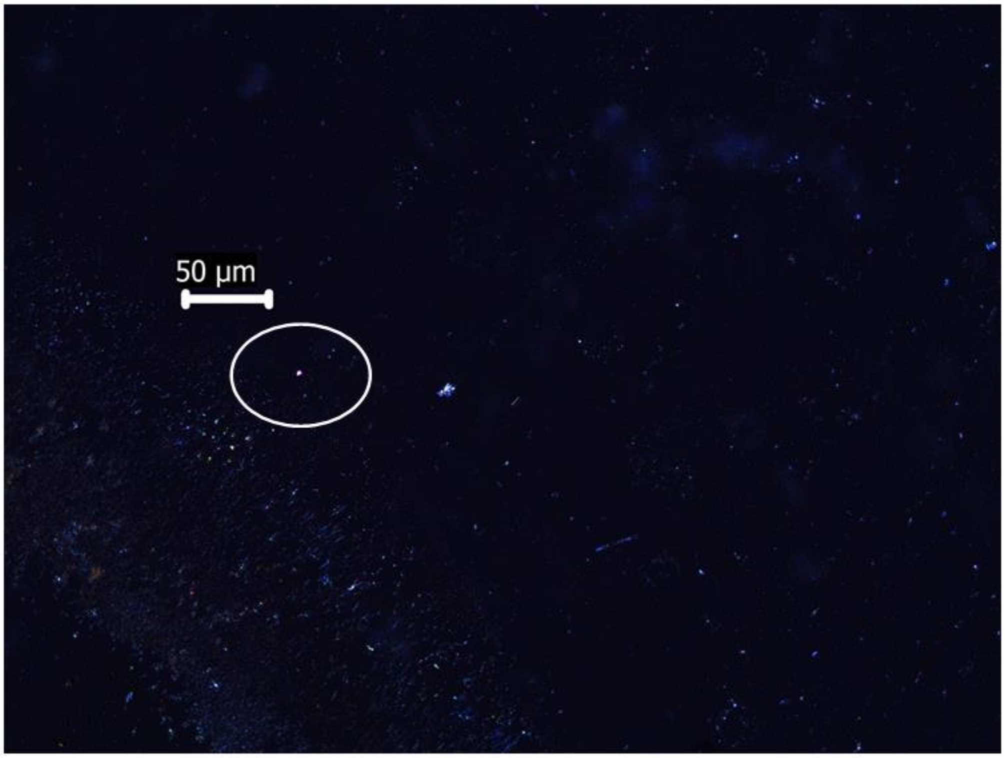

S11-d (α = 135°) maximum light intensity

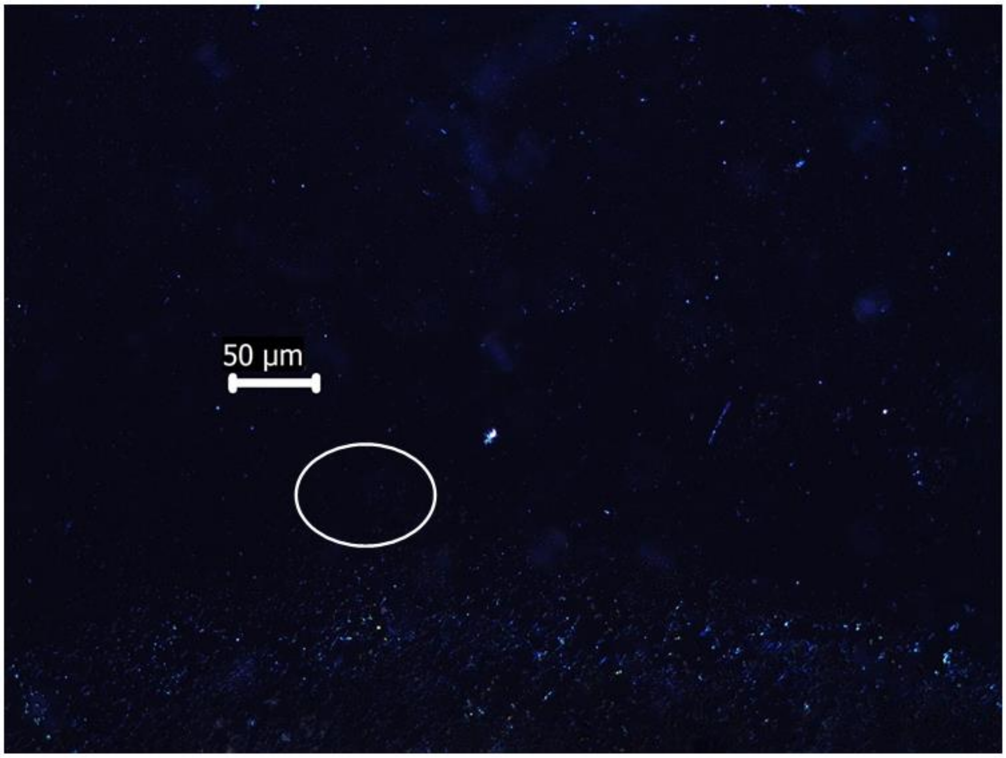

S11-e (α = 180°) extinction

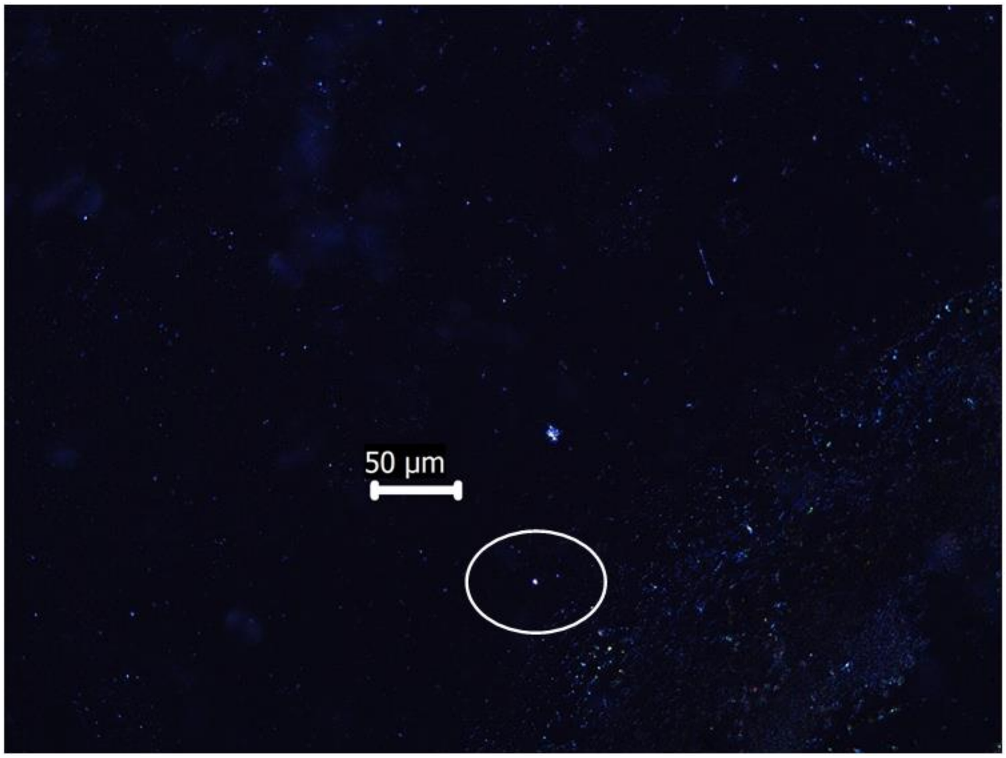

S11-f (α = 225°) maximum light intensity

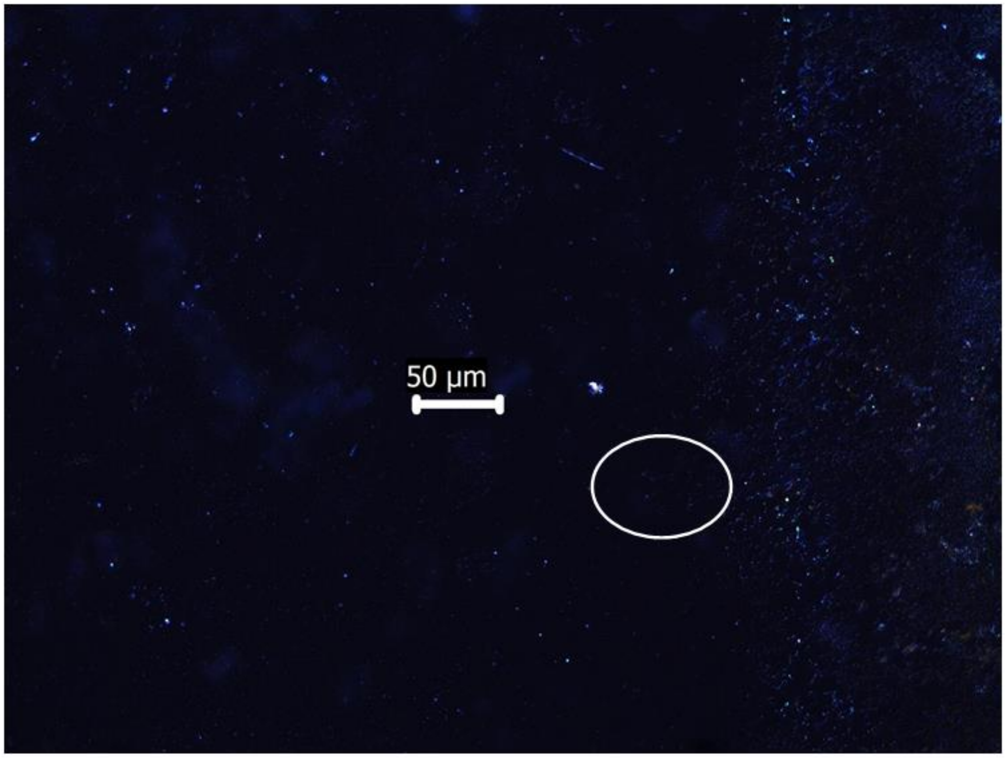

S11-g (α = 270°) extinction

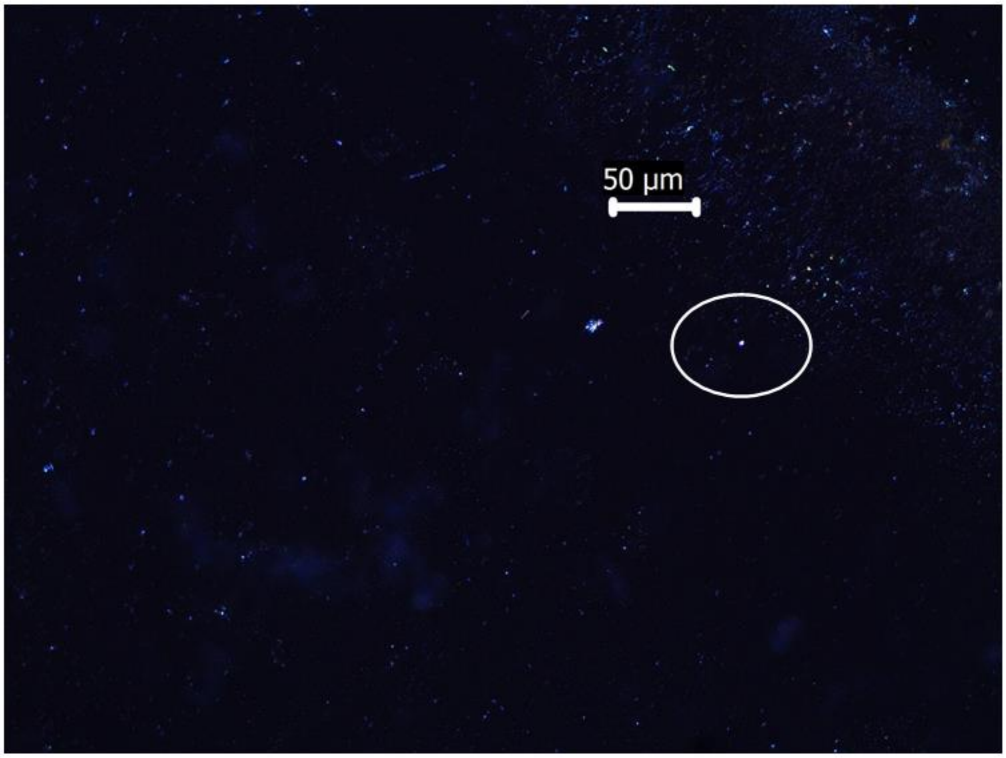

S11-h (α = 315°) maximum light intensity

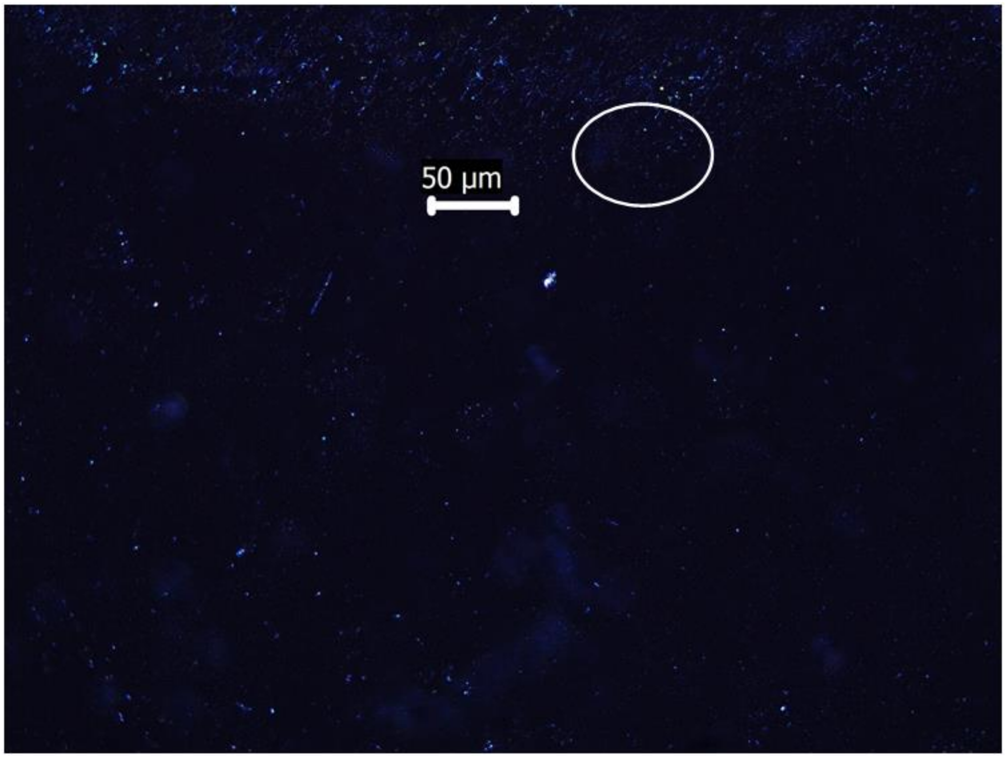

S11-i (α = 360°) extinction
